# QSAR Modeling and Fragment Contribution Analysis of N-Benzyltryptamines at Human 5-HT2 Receptor Subtypes

**DOI:** 10.3390/molecules31203764

**Published:** 2026-10-20

**Authors:** Vladimir Đorđević, Iva Binić, Jovana Petrović, Jelena Stojanov, Žarko Mitić, Aleksandar M. Veselinović

**Affiliations:** 1 Department of Psychiatry with Medical Psychology, Faculty of Medicine, University of Niš, 18000 Niš, Serbia; vlajce74.vdj@gmail.com; 2 Clinic of Psychiatry, University Clinical Center Niš, 18000 Niš, Serbia; ivabinic@gmail.com (I.B.); jovanapetrovic0912@gmail.com (J.P.); 3 Center for Mental Health Protection, University Clinical Center Niš, 18000 Niš, Serbia; jelena.a.86.ja@gmail.com; 4 Department of Chemistry, Faculty of Medicine, University of Niš, 18000 Niš, Serbia; zarko.mitic@medfak.ni.ac.rs

**Keywords:** 5-HT2 receptors, N-benzyltryptamines, quantitative structure-activity relationships, simplex descriptors, model interpretation, applicability domain, non-additivity, subtype selectivity

## Abstract

Serotonin 5-HT2 receptor agonists are returning to psychiatric drug development, but separating the three receptor subtypes pharmacologically remains difficult, and agonism at 5-HT2B carries a risk of valvular heart disease. A recent study reported affinities of 46 N-benzyltryptamines and N-benzyl-5-methoxytryptamines at all three human subtypes and analyzed them with an additive substituent model, which gave fragment coefficients of the same sign at every subtype and suggested that selectivity within this scaffold would be hard to achieve. We revisited that conclusion with non-additive models built under a protocol designed for small datasets: fifty stratified partitions with all filtering and hyperparameter tuning inside each training set, external statistics reported as distributions, response permutation on three partitions per endpoint, and partitions that withhold an entire scaffold or an entire substitution position. We repeated the analysis with two descriptor sets that share no variables and are built on different principles. Penalized linear models were predictive and transferable, whereas gradient boosting reproduced the training set exactly, and neither tree ensemble transferred to a held-out scaffold. Fragment contributions at 5-HT2A and 5-HT2C correlated at 0.25 compared with 0.77 for whole-molecule affinities, and both descriptor sets gave the same figure within 0.01. All models failed on para-substituted analogs, which distance-based applicability-domain criteria did not flag, whereas models trained without the ortho or the meta block stayed above a mean-predictor baseline. Aggregation over the whole molecule, rather than descriptor choice, conceals subtype divergence. One group drives the divergence: the N-benzyl substituent raises the predicted 5-HT2A affinity and lowers the predicted 5-HT2C affinity; excluding it raised the correlation to 0.93.

## 1. Introduction

The serotonin 5-HT2 receptor family has returned to the center of psychiatric drug discovery after two decades on the margins. A single 25 mg dose of psilocybin, whose active metabolite, psilocin, is a 5-HT2A receptor agonist, produced a statistically and clinically significant reduction in depression scores in a phase 2b trial of 233 patients with treatment-resistant depression [1]. Comparable signals have been reported for related agonists in anxiety, substance use disorder, and end-of-life distress. The 5-HT2A receptor is widely considered the molecular entry point for these effects: the head-twitch response in rodents and the subjective effects in humans are blocked by 5-HT2A antagonists, and the sustained promotion of cortical spinogenesis, thought to underlie the antidepressant response, proceeds through 5-HT2A together with TrkB, mTOR, and AMPA receptor signaling [2,3].

This creates an unusual problem in medicinal chemistry because the therapeutic idea and the principal liability of the chemotype are linked to the same receptor, prompting the search for ligands that dissociate the two effects. Compounds such as tabernanthalog, IHCH-7079, IHCH-7086, and the (R)-69 and (R)-70 series have been reported as 5-HT2A agonists that promote neural plasticity in rodents while producing little or no head-twitch response, and are commonly described as non-hallucinogenic agonists or psychoplastogens [3,4]. That description should be read with care. The head-twitch response is the standard preclinical proxy for hallucinogenic potential, but its absence in rodents does not by itself establish the absence of psychedelic effects in humans, and for none of these compounds has the relationship between the two been established clinically [5]. How the apparent dissociation arises is also unsettled. Pharmacological evidence indicates that signaling efficacy rather than biased agonism separates psychedelic from non-psychedelic 5-HT2A agonists, and that several compounds reported as biased are better described as weak partial agonists [5,6], while other explanatory models remain under discussion. What matters for the present work is that the question is open and being pursued within a chemotype whose subtype pharmacology is itself unresolved. Progress therefore depends on understanding the pharmacology of closely related receptors, which shifts the problem from the clinic to the chemistry.

The three 5-HT2 subtypes are very similar in their orthosteric pockets but have very different therapeutic consequences. 5-HT2C agonists reduce appetite via pro-opiomelanocortin neurons and have been explored for addiction and psychosis; lorcaserin was approved on that basis in 2012 and withdrawn in 2020 after a cardiovascular outcome trial indicated an imbalance in cancer diagnoses [7,8]. 5-HT2B is an antitarget. Its activation stimulates mitogenic growth of valve interstitial cells and is causally linked to valvular heart disease associated with fenfluramine and its metabolite norfenfluramine, as well as pergolide, cabergoline, and methysergide, a mechanism that led to fenfluramine’s withdrawal in 1997 [9,10,11]. Any serotonergic agonist intended for clinical use must therefore be assessed for 5-HT2B activity, and a compound that combines 5-HT2A potency with 5-HT2B agonism has not meaningfully improved.

N-benzylation is one of the most effective potency-enhancing modifications reported in this chemical space and also influences subtype selectivity. Adding an N-benzyl group to phenethylamines in the 2C series increases 5-HT2A affinity by one to two orders of magnitude, producing compounds such as the NBOMe and NBOH series [12,13]; the same modification produces comparable gains in tryptamines [14]. Structural studies have begun to clarify the basis for this effect. In the cryo-electron microscopy structure of the Gq-coupled 5-HT2A receptor bound to 25CN-NBOH, the N-benzyl group extends into a pocket beyond the orthosteric site [15]. This pocket is one of the least conserved regions between 5-HT2A and 5-HT2C and includes positions where the receptors differ, including one where a leucine in 5-HT2A is replaced by a valine in 5-HT2C [16]. Selectivity is unlikely to depend on any single substitution. Recent structure-guided work using this same N-benzyl scaffold achieved high 5-HT2A over 5-HT2C selectivity by targeting a different position, a leucine in transmembrane helix 2 of the extended pocket, and confirmed the result against 5-HT2C RNA-editing isoforms, reciprocal mutants and rodent orthologues [16]. Whatever its structural basis, the benzyl ring is the part of the molecule that reaches this divergent region. Substituent effects on that ring are therefore where subtype divergence should be sought, and a complete congeneric series across the ring provides the dataset to do so. The chemistry thus points to the benzyl ring, and the question that follows is whether the statistical model used to interpret that chemistry can reveal divergence at all.

Martínez-Afani and colleagues recently reported a series of 46 N-benzyltryptamines and N-benzyl-5-methoxytryptamines with pKi values across all three human 5-HT2 subtypes, designed as a complete factorial combination of two scaffolds, seven substituents, and three ring positions [17]. Complete factorial designs are uncommon in medicinal chemistry and are far more informative than opportunistically assembled sets because they separate substituent effects from scaffold effects and allow additivity to be tested directly. The authors analyzed the data using Free–Wilson analysis [18] and found that fragment coefficients had the same sign across all three subtypes, suggesting that selectivity within this scaffold and avoidance of 5-HT2B liability in particular would be difficult to achieve.

This conclusion raises a methodological question rather than a chemical one. Free–Wilson analysis assumes that substituent effects are additive and independent of context. This assumption fails in many congeneric series, with deviations large enough to be termed additivity cliffs, by analogy with activity cliffs [19,20]. When additivity does not hold, an additive model does not report the failure; it distributes the discrepancy evenly among its coefficients and returns a plausible fit. A model that cannot represent interactions between substituents cannot, by construction, show whether fragment effects differ between receptors. Whether the observed similarity of fragment effects across the three subtypes reflects the receptors or the model therefore remains open, and this dataset makes it answerable.

Addressing this question requires careful considerations that are easy to overlook at this sample size. With 46 compounds and over a thousand candidate descriptors, a single train–test split offers limited information, and filtering descriptors on the entire dataset can leak test-set data into the model. Additionally, internal fit statistics may seem excellent even without real predictive power [21,22]. Conclusions based on one descriptor system might reflect that specific system rather than the underlying data, a scenario rarely tested because a second, unrelated descriptor dictionary is seldom computed. To address these issues, all filtering and selection are conducted within the training set of each of the fifty repeated stratified splits. The Golbraikh–Tropsha and Roy acceptance criteria are reported as distributions rather than single best values [23,24,25], and the entire analysis is repeated using a second descriptor dictionary on identical data partitions, allowing for paired comparisons.

The two dictionaries differ in their unit of description. The simplex representation encodes a molecule as counts of tetratomic fragments labeled by partial charge, lipophilicity, refractivity, or hydrogen-bonding character [26,27,28], so models built on it are local and fragment-based. PaDEL descriptors are continuous properties of the whole molecule, including constitutional, topological, autocorrelation, and electrotopological indices [29]. In both cases, structural fragments are interpreted using the fragment-removal approach of Polishchuk and colleagues [30,31], which isolates the contribution of a structural element from any model, regardless of the descriptors or the learning algorithm. This allows us to compare the two dictionaries not only on predictive performance, but also on what they say about the molecules.

This work has three objectives. First, we determine whether the similarity of fragment effects across 5-HT2A, 5-HT2B, and 5-HT2C reflects the receptors or the additive analysis by interpreting validated non-additive models at the fragment level. The second is to test whether that answer holds under a change in descriptor representation by repeating the analysis with a second dictionary over the same partitions. The third is to identify where these models are reliable and where they are not, using partitions that withhold an entire scaffold or an entire substitution position, and asking whether routine applicability-domain criteria anticipate the failures. These findings inform how analyses of this kind should be run before proposing compounds, which we view as more useful to the field than another list of predicted analogs.

## 2. Materials and Methods

### 2.1. Dataset

Affinity data were taken from Martínez-Afani et al. [17], who reported pKi values for 46 compounds at the human 5-HT2A, 5-HT2B, and 5-HT2C receptors: 22 N-benzyltryptamines, 22 N-benzyl-5-methoxytryptamines, and the parent amines tryptamine and 5-methoxytryptamine. Affinities were measured by radioligand displacement using [3H]ketanserin (5-HT2A), [3H]LSD (5-HT2B), and [3H]mesulergine (5-HT2C) in three independent experiments. The series comprises a complete factorial combination of two scaffolds, seven benzyl substituents (OMe, Me, Br, Cl, I, F, NO_2_), and three ring positions (ortho, meta, para), together with the unsubstituted benzyl analogues and the two parent compounds.

Five endpoints were modeled: pKi at each of the three receptors, and the two difference endpoints ΔpKi(2A–2C) and ΔpKi(2A–2B). We did not model functional data from the source study (pEC50 values from calcium mobilization) because 22 out of 138 values are censored, and censoring is associated with low activity, so removing them would bias the training set toward active compounds.

Structures were not transcribed. SMILES were automatically generated from a fixed scaffold template and a substituent token, then verified through seven independent checks: RDKit 2026.03.5 parsing; comparison of the molecular formula from the SMILES with that from the compound name in the source paper; verification of the ortho, meta, or para pattern by graph distance within the benzyl ring; comparison of the exact [M + H]^+^ mass with the HRMS calculated values reported for the five newly synthesized compounds; uniqueness of all 46 InChIKeys; confirmation that the indole methoxy group is at C5 via substructure matching against an independently written reference structure; and internal consistency of the transcribed pKi values with the reported Ki values in nM. All checks passed. Structures are treated as free bases, with counter-ions (hydrochloride or fumarate for the two parent compounds) removed, as is standard for descriptor calculation. Generation and validation scripts are provided in the Supplementary Materials.

### 2.2. Descriptor Calculation

#### 2.2.1. Simplex Descriptors

Two-dimensional simplex descriptors were computed using the open-source simplex representation of molecular structure (SiRMS 1.1.1, accessed through the sirms 1.2.4 Python package) [26,27,28]. A simplex is a tetratomic fragment with fixed composition and connectivity, and a simplex descriptor counts how often a given simplex occurs in a molecule. Each vertex can carry an atomic property label, allowing the same topological fragment to be distinguished by the physicochemical nature of its atoms.

We used fragments consisting of exactly four atoms (min_atoms = max_atoms = 4), allowing one or two disconnected components (min_components = 1, max_components = 2), with hydrogen atoms retained and descriptors expressed as fragment counts. We applied six vertex-labeling schemes: element, topology (unlabeled), partial charge, lipophilicity, refractivity, and hydrogen-bond character. This produced 1664 raw descriptors.

We computed the atomic properties required by the four physico-chemical schemes using RDKit [32] and supplied them to SiRMS as SDF fields. Partial charges were Gasteiger-Marsili charges [33]; atomic lipophilicity and molar refractivity were Crippen atomic contributions [34]; hydrogen-bond character was assigned via SMARTS matching with standard donor and acceptor definitions, with each atom labeled as donor, acceptor, both, or neither. We discretized continuous properties at boundaries specified in the SiRMS setup file: −0.05, 0, and 0.05 for partial charge; −0.2, 0, and 0.2 for lipophilicity; and 1.5, 2.5, and 3.5 for refractivity. These boundaries are generic rather than those used in earlier simplex studies, which is acknowledged as a limitation because it prevents direct numerical comparison of individual descriptors with the published simplex literature.

#### 2.2.2. PaDEL Descriptors

Two separate descriptor sets were generated using PaDEL-Descriptor 2.21 [29] via the padelpy 0.1.17 interface as an independent group. The process included salt removal, nitro-group standardization, and aromaticity detection, while three-dimensional descriptors and fingerprints were disabled, and the input order was preserved. This resulted in 1444 descriptors with no missing values. The two dictionaries contain entirely different variables: they do not share any descriptor names, definitions, or construction rules. They also differ in measurement units: PaDEL descriptors are continuous properties calculated over the whole molecule, including constitutional, topological, autocorrelation, electrotopological-state, BCUT descriptors, and physico-chemical properties. In contrast, simplex descriptors are sparse counts of local fragments. Although both sets were computed for the same 46 molecules, they are likely to be numerically related to some extent. Therefore, the redundancy between them was assessed rather than assumed, both for the raw dictionaries and the descriptors actually used in the models (see Section 3.9).

### 2.3. Training and Test Partitions

All models were built using repeated random splits rather than a single split, and the reported statistics are distributions across repeats rather than a best-case value, following recommendations for small datasets [21,35]. We generated fifty stratified 75/25 partitions, with 34 compounds for training and 12 for testing, and kept the training set size constant across repeats.

Two stratification strategies were analyzed. In design A, strata consisted of quartiles of the modeled response. For all models reported, we used design B, dividing samples into eight groups by scaffold (tryptamine or 5-methoxytryptamine) and benzyl substitution position (ortho, meta, para, or unsubstituted), ensuring that each substitution pattern appeared in both partitions. A three-way stratification including activity was not feasible because it would produce thirteen strata for a test set of only twelve. To assess activity balance empirically, we found a mean deviation of 0.08 log units from the overall mean. Because stratification depends solely on structure rather than descriptors or response, we reproduced the same partitions across both descriptor sets, allowing every comparison to be paired.

Partitions were created using scikit-learn 1.8.0 stratified shuffle split with a fixed seed, ensuring that all fifty were deterministic and reproducible from the deposited scripts. Compounds were assigned to the eight specified strata, and within each, the assignment was random with a test fraction of 0.25. This approach guarantees that every scaffold and ring position appears in both partitions across all repeats. A deterministic alternative was also tested. The Kennard–Stone algorithm was used to select a training set that evenly covered the descriptor space, applied separately in each space after removing constant and collinear descriptors and without considering the response, resulting in 34 training compounds and 12 test compounds.

Structured partitions served as stress tests. In the scaffold-out partition, the 23 tryptamine analogs formed the training set and the 23 5-methoxytryptamine analogs the test set. In the position-out partitions, we withheld one substitution position at a time: the training set comprised the compounds carrying the two remaining positions, together with the two parent amines and the two unsubstituted benzyl analogs (32 compounds), and the test set comprised the 14 compounds of the withheld position. We performed filtering, standardization, hyperparameter selection, and applicability-domain calculation exactly as for the random partitions, and exclusively within the training block. Because the three positional blocks lie at different distances from the training mean, performance on each was also compared to a null model that returns the training-set mean for every test compound; the ratio of the model RMSE to the null RMSE separates a genuine loss of predictive information from the arithmetic effect of predicting a displaced block [36]. For the observed-versus-predicted analysis, we ran twelve repeats of stratified fourfold cross-validation, preserving the 75/25 ratio and yielding twelve external predictions for each compound.

### 2.4. Descriptor Filtering

Filtering was performed independently within the training partition of each repeat and never on the full dataset, so information from test compounds could not influence any modeling decision. For simplex descriptors, the steps were the removal of constant descriptors, removal of descriptors with fewer than four non-zero values in the training set, prescreening to the 300 descriptors most correlated with the training response, and greedy removal of descriptors correlated above |r| = 0.95 with an already retained descriptor, keeping the one more strongly correlated with the response. This left a median of 94 to 112 descriptors, depending on the endpoint. We applied the same steps to the PaDEL descriptors, except for the minimum-non-zero rule, which applies to fragment counts but not continuous variables, leaving a median of 127 to 165 descriptors. Descriptors were then standardized to zero mean and unit variance using statistics computed on the training partition alone.

The prescreened pool size is an adjustable parameter within the protocol. A larger pool might enable a model to form from descriptors that, by chance, correlate with the response, an effect that is more likely as the sample size decreases [37]. Therefore, we empirically tested its impact by repeating the entire protocol after reducing the pool to 300, 100, 50, and 25 descriptors, while keeping all other settings the same.

### 2.5. Modeling Algorithms

The algorithms were selected not to maximize performance but to span a ladder of model complexity on a sample of 46 compounds. Multiple linear regression is the classical QSAR reference and the form in which the original additive analysis was cast. PLS and the penalized linear models address the collinearity that necessarily arises when descriptors outnumber compounds, but through different regularization geometries: PLS projects onto latent variables, ridge shrinks all coefficients, lasso selects among them, and elastic net combines the last two. We included support vector regression and the two tree ensembles as non-linear references, since linear models alone cannot settle a question about non-additivity. The ladder is diagnostic rather than competitive: a flexible learner that reproduces the training set exactly and then fails to transfer is itself a result, and is reported as such in Section 3.3 rather than concealed by reporting only the best model.

We applied eight algorithms to each partition and endpoint using scikit-learn [38], except for gradient boosting, which employed XGBoost 3.4.1 [39]. We optimized hyperparameters through fivefold cross-validation within the training partition. Multiple linear regression used forward selection among the sixty descriptors most correlated with the response, adding up to four variables and accepting a variable only if it increased the leave-one-out Q2 of the training set, calculated analytically from the hat matrix. Partial least squares employed between one and six latent variables, selected via inner cross-validation. Ridge regression was fitted across forty penalties logarithmically spaced from 1 × 10^−2^ to 1 × 10^4^, while lasso utilized sixty automatically chosen penalties. Elastic net used forty penalties with L1 ratios of 0.2, 0.5, 0.8, and 0.95. Support vector regression used a radial basis kernel with C values of 1, 10, and 100; gamma set to the scale heuristic, 0.01, and 0.1; and epsilon of 0.05, 0.1, and 0.2. We trained the random forest with 500 trees, requiring at least two samples per leaf and considering 30% of descriptors at each split. Gradient boosting used 400 trees of depth three, a learning rate of 0.05, subsampling at 0.8, column subsampling at 0.5, and an L2 penalty of 2.

Because penalized models have no exact parameter count, an effective number of parameters was computed for the statistics that require one: the trace of the hat matrix, derived from the singular values of the standardized training matrix and the selected penalty for ridge; the number of non-zero coefficients for lasso and elastic net; the number of latent variables for PLS; and the number of retained descriptors for the non-parametric methods. The F statistic and the standard error of the estimate were derived using that value.

### 2.6. Validation Statistics

Internal assessment included the coefficient of determination and its adjusted form, the F statistic, and the standard error of the estimate. External statistics were calculated on the held-out compounds of each partition and included the test-set coefficient of determination, which, by definition, aligns with Q2F2. We also calculated Q2F1, the predictive squared correlation coefficient, which relates the residual sum of squares to the training-set mean and is therefore more stringent [40,41]. Additional metrics included the root mean square error, mean absolute error, maximum absolute error, and mean signed error. To evaluate agreement between the observed and predicted values, we used the concordance correlation coefficient, which accounts for both systematic shift and scale error and serves as the most rigorous single external criterion [25].

The Golbraikh–Tropsha criteria were evaluated in full [21,23]: the squared correlation coefficient of the test set, the squared correlation coefficients of the two regressions through the origin, the slopes k and k’, and the relative difference (r2 − r02)/r2. Acceptance requires r2 above 0.6, a relative difference below 0.1, and both slopes between 0.85 and 1.15. We computed the rm2 metrics of Roy and colleagues in both directions and report their average and absolute difference, with acceptance at rm2 above 0.5 and Δrm2 below 0.2 [24,42]. All statistics are reported as medians over fifty repeats, with interquartile ranges and the proportion of repeats exceeding a Q2F1 of 0.5.

### 2.7. Y-Randomization

We evaluated chance correlation by response permutation [22] across three partitions for each endpoint rather than a single one. The three partitions were those whose ridge Q2F1 was closest to the lower quartile, median, and upper quartile of the distribution over the fifty repeats, so the test is not conditioned on a single favorable or unfavorable split. We used 50 permutations on the median partition of each endpoint with the simplex dictionary, and 30 on the remaining partitions and throughout for PaDEL, so the empirical *p*-value is bounded below by 0.02 and 0.033, respectively. For each permutation, we repeated the full modeling procedure, including hyperparameter selection, and recorded internal and external statistics. We computed cRp2 as R times the square root of the difference between the true R2 and the mean R2 of the permuted models; we accepted values above 0.5, and we defined the empirical *p*-value as the proportion of permutations in which external Q2F1 reached or exceeded that of the unpermuted model.

### 2.8. Applicability Domain

We defined the applicability domain in the standardized descriptor space of each training partition as the distance to the five nearest training compounds. The acceptance threshold was the mean plus three standard deviations of those distances within the training set, and results are expressed as the ratio of a test compound’s distance to that threshold, with values below one indicating that the compound lies within the domain. Leverage-based Williams analysis was not used because, with more retained descriptors than training compounds, the hat matrix is degenerate and leverage values are not informative for the penalized models employed here.

### 2.9. Fragment Contributions

Structural interpretation used the fragment-removal method of Polishchuk and colleagues [30,31,43], in which the contribution of a structural element is the difference between the prediction for the whole molecule and the prediction for the molecule lacking that element. Two levels were used. At the atomic level, which applies only to simplex models, SiRMS generated per-atom descriptor sets in per-atom-fragment mode, excluding from the count every simplex containing the target atom. At the fragment level, which applies to both dictionaries, we built three truncated molecules per compound with RDKit by removing the benzyl-ring substituent, the indole 5-methoxy group, and the entire N-benzyl group, yielding 108 truncated structures. We recomputed descriptors for the truncated molecules from scratch in both dictionaries using identical settings, so the two interpretations are directly comparable.

We computed contributions using the ridge ensemble, and for each compound, we used only models in which that compound was in the test partition, so no contribution came from a model that had seen the compound during training. This is more conservative than the common practice of interpreting a single model fitted to all data. Because successive removal of structural elements causes overlapping descriptors to be counted more than once, absolute contributions are not meaningful, and the analysis is restricted to rankings, differences within a receptor, and correlations between contribution profiles.

Stratification by scaffold and ring position places the two parent amines and the two unsubstituted benzyl analogs in strata of two members each, so a test partition of twelve never includes them. Under the fifty repeated splits, these four compounds received no contribution, and three of the 108 truncated structures, all belonging to the two unsubstituted benzyl compounds, lacked an estimate. We therefore also computed contributions under the twelvefold repeated stratified fourfold cross-validation of Section 2.3, in which we held out each compound twelve times, so that all 46 compounds and all 108 truncations were covered. Both sets are reported.

Because a contribution is the difference between two model predictions, it carries model error twice, whereas an experimental affinity carries only measurement error. Therefore, correlations computed at the two levels are not automatically comparable. The reliability of each was therefore estimated to disattenuate both: for the contributions, from the standard error of each estimate across models in which the parent compound was held out, relative to the variance between fragments; and for the experimental affinities, from the replicate standard deviations reported in the source study for three independent determinations.

### 2.10. Ablation and Selection Stability

We assessed the contribution of each simplex labeling scheme by rebuilding ridge models using that scheme alone and by combining the two topological schemes with the four physico-chemical schemes, using the same partitions and filtering procedure. Selection stability was measured by counting, over the fifty repeats, how often each descriptor received a non-zero lasso coefficient and noting the sign. Descriptors selected in at least 40% of repeats are reported, along with the consistency of their sign.

### 2.11. Statistical Comparison of the Two Dictionaries

Because the partitions are numerically identical across the two descriptor dictionaries, we compared them pairwise. For each algorithm and endpoint, we tested the fifty paired differences in Q2F1 using the Wilcoxon signed-rank test as implemented in SciPy 1.17.1 [44] and reported the median difference and the proportion of partitions favoring each dictionary as the primary measure of effect. Forty comparisons were made, so raw *p*-values were adjusted for multiplicity using the Benjamini–Hochberg procedure at a 5% false discovery rate [45], and the more conservative Holm-adjusted values are reported alongside.

Repeated random partitions of a single dataset are not independent experiments. On average, any two of the fifty training sets share about two thirds of their compounds, and a paired test that ignores this overlap underestimates the variance of the difference and therefore overstates significance [46]. As a sensitivity analysis, we also computed the corrected resampled *t*-test of Nadeau and Bengio, inflating the variance of the paired differences by 1/k + n_test/n_train, which equals 0.373 for fifty partitions of 34 training and 12 test compounds. Accordingly, the comparison of the two dictionaries is presented as a comparison of effect sizes, with statistical significance treated as supporting rather than load-bearing evidence.

### 2.12. Software and Reproducibility

All calculations were performed in Python 3.12 with RDKit 2026.03.5, SiRMS 1.1.1 through the sirms 1.2.4 Python package, and PaDEL-Descriptor 2.21 through padelpy 0.1.17, together with scikit-learn 1.8.0, XGBoost 3.4.1, SciPy 1.17.1, pandas 3.0.5 and NumPy 2.5.3. A single random seed, 20260902, was used for dataset partitioning and for all stochastic algorithms. All scripts needed to reproduce the dataset, descriptors, models, validation statistics, fragment contributions, and figures are provided as Supplementary Materials and in the deposited archive.

## 3. Results

### 3.1. Dataset, Descriptor Generation and Split Design

The dataset comprises 46 compounds reported by Martínez-Afani et al. [17], including 22 N-benzyltryptamines, 22 N-benzyl-5-methoxytryptamines, and two parent amines, each characterized by pKi values at the human 5-HT2A, 5-HT2B, and 5-HT2C receptors. We generated structures automatically from a fixed scaffold and a substituent token rather than transcribing them by hand, and passed seven independent consistency checks (Supplementary Table S1). The most informative of these is external: the exact [M + H]^+^ masses computed from the structures match the five HRMS-calculated values listed in the original paper to four decimal places.

The series is a full factorial design across two scaffolds, seven substituents, and three ring positions. Such comprehensive designs are uncommon in medicinal chemistry datasets; they separate substituent effects from scaffold effects and allow for a direct evaluation of additivity, as in Section 3.6 and Section 3.7.

We generated two-dimensional simplex descriptors using six atom-labeling schemes (element, topology, partial charge, lipophilicity, refractivity, and hydrogen-bond character), yielding 1664 raw descriptors [26,27]. All filtering was performed within each training partition during resampling: constant descriptors were removed, descriptors with fewer than four non-zero values were excluded, the remainder were prescreened to the 300 most correlated with the response, and descriptors with |r| > 0.95 were pruned greedily. This left a median of 94 to 112 descriptors, depending on the endpoint.

Two split designs were compared. In design A, the 75/25 split was stratified by activity quartile alone; in design B, by scaffold and ring position, so that every substitution pattern appeared in both partitions. Design B was used for all reported analyses. At the three affinity endpoints, the two designs performed similarly, with median differences in external predictivity ranging from −0.065 to +0.016, indicating that the results are not an artifact of the partition. At the two selectivity endpoints, design B showed a median decrease of 0.09 to 0.24 because stratification by activity alone allowed test sets to omit entire ring-position classes, which carry the selectivity signal (Section 3.7).

### 3.2. Model Performance

**Figure 1:**
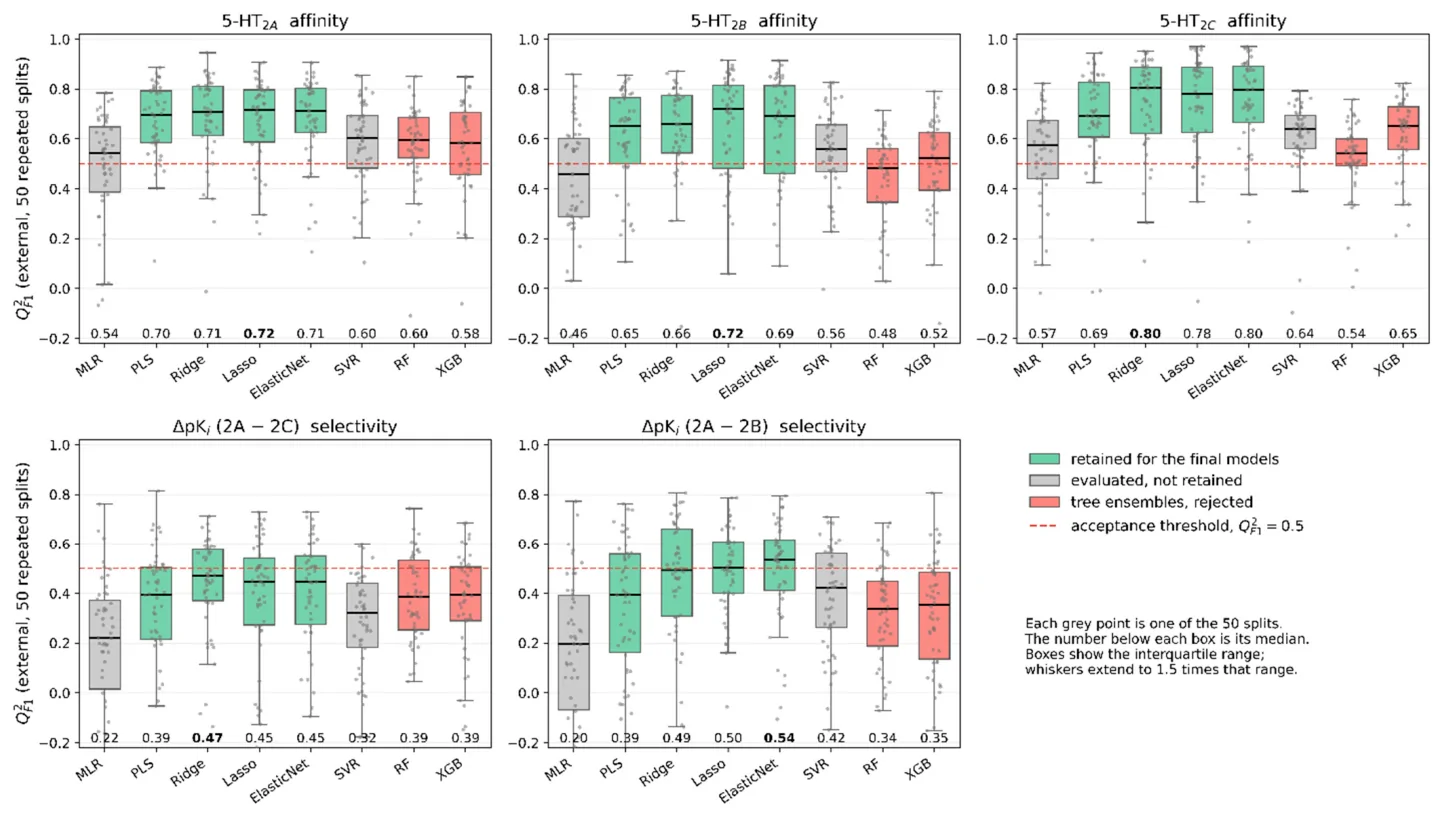
External predictivity (Q^2^F1) of the eight algorithms at five endpoints over 50 stratified 75/25 splits. Gray points are individual splits, medians are printed below each box, bold for the best model per endpoint. Green, retained; gray, not retained; red, tree ensembles (Section 3.3). Dashed line, acceptance threshold 0.5.

Eight algorithms were trained on 50 repeated splits per endpoint. Because a single split provides very little information at this sample size, the distribution of external statistics is reported rather than a single best case (Figure 1). Table 1 presents median statistics for the four linear models at the three affinity endpoints; all eight algorithms and all five endpoints, with interquartile ranges, are in Supplementary Table S2.

Figure 1 shows the distribution of external predictivity (Q2F1) across 50 stratified 75/25 splits under design B for eight algorithms and five endpoints. Each gray point represents one split, and the median is shown below each box; the best model for each endpoint is in bold. Green indicates models retained for the final analysis, gray indicates models evaluated but not retained, and red indicates tree ensembles rejected for the reasons given in Section 3.3. The dashed line is the conventional acceptance threshold of 0.5. Both selectivity endpoints, in the lower row, fell below that threshold for every algorithm.

All three affinity models met the conventional acceptance criteria [23,24,25]: R^2^tr above 0.6, Q2F1 above 0.5, R^2^te above 0.6, r^2^m above 0.5, and Δr^2^m below 0.2. The slopes k ranged from 0.998 to 1.007, and (r^2^ − r0^2^)/r^2^ from 0.014 to 0.049, so the Golbraikh–Tropsha conditions were satisfied with a wide margin. Δr^2^m ranged from 0.126 to 0.187, which passed but with less margin than the other criteria. We state this explicitly here rather than leaving it to the Supplementary Table S2.

**Figure 2:**
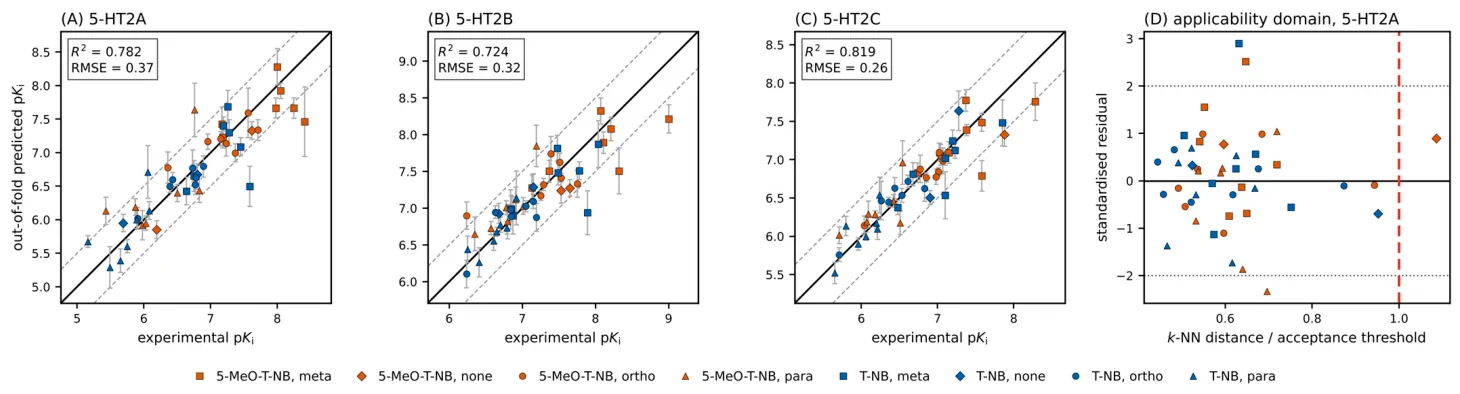
Out-of-fold predictions from twelve repeats of stratified fourfold cross-validation with ridge regression. (**A**–**C**) Experimental versus predicted pKi at the three receptors; error bars, SD of the twelve predictions; solid line, identity; dashed lines, ±0.5 log units. (**D**) Standardized residual versus k-nearest-neighbor distance normalized by the acceptance threshold (red line), for the same three receptors. Symbols, benzyl position; colors, scaffold. The criterion places 98%, 100%, and 98% of compounds within the domain, including every para-substituted analog.

Out-of-fold predictions from twelve repeats of stratified fourfold cross-validation, which preserves the 75/25 ratio and provides an external prediction for every compound, yielded R^2^ values of 0.782, 0.724, and 0.819 and RMSE values of 0.37, 0.32, and 0.26 log units at 5-HT2A, 5-HT2B, and 5-HT2C (Figure 2). The mean absolute errors of 0.32, 0.25, and 0.20 log units are close to the propagated experimental uncertainty of about 0.2 log units, indicating that the models are approaching the data’s resolution.

Ridge retained a median of 18 to 21 non-zero effective parameters across 34 training compounds, so an R^2^tr near 0.95 is not an unconstrained fit. We performed response permutations on three partitions per endpoint, selected from the lower quartile, median, and upper quartile of the ridge Q2F1 distribution (Table 2 and Supplementary Table S3). Across all combinations of dictionary, endpoint, partition, and algorithm, cR^2^p ranged from 0.56 to 0.94, above the 0.5 threshold, and the empirical *p*-value was at its lower bound throughout: *p* ≤ 0.02 with fifty permutations and *p* ≤ 0.033 with thirty. For ridge and lasso, the median permuted Q2F1 ranged from −0.02 to +0.01, regardless of whether the partition was poor, typical, or good; for PLS, it was systematically negative, between −0.19 and −0.56. The highest external Q2F1 reached by any permuted model was 0.625, against a lowest unpermuted value of 0.570, so the two distributions touched at one point and did not overlap. Individual permutations reached a scrambled R^2^tr as high as 0.99 while their external Q2F1 remained at zero, which is the clearest single demonstration that internal fit alone establishes nothing.

One qualification applies to these numbers. Under a permuted response, the lasso shrinks to the intercept, so its scrambled R^2^ and Q2F1 are zero by construction rather than because of the absence of chance correlation, and ridge behaves similarly at high penalty. The informative case is PLS, which retains a fixed number of latent variables and can therefore fit a permuted response; the argument rests on the contrast between its scrambled internal fit and its negative external predictivity.

The reported performance also does not depend on the size of the candidate descriptor pool. Reducing univariate prescreening from 300 to 100, 50, and 25, with all other settings unchanged, gradually lowers external predictivity rather than abruptly (Table 3). At a pool of 25, the models retained a median of 16 to 20 descriptors for 34 training compounds; that is, fewer variables than half the sample, and the median Q2F1 remained 0.43 to 0.67. More informative than the level is the distance between internal fit and external predictivity. The difference between R^2^tr and Q2F1 remained between 0.18 and 0.28 across a twelvefold change in pool size, and at 5-HT2C, it narrowed rather than widened, from 0.27 to 0.18. If the larger pool had been buying fit on noise, internal fit would have risen while external predictivity would have fallen; instead, both rose together.

We also tested a random stratified design rather than a deterministic one. A Kennard–Stone split, evaluated against the distribution of the fifty stratified partitions, behaved differently across the two descriptor spaces (Supplementary Table S17). With the simplex dictionary, it lay above the median at every endpoint, and for every penalized model, between the 52nd and 86th percentiles, yielding Q2F1 values of 0.765, 0.782, and 0.820 at 5-HT2A for PLS, ridge, and lasso, compared with medians of 0.696, 0.708, and 0.716. With the PaDEL dictionary, it lay above the median in six out of the nine combinations and failed outright in one: PLS at 5-HT2C, yielding 0.113 against a median of 0.719. The same deterministic procedure applied to the same 46 compounds therefore yields an estimate anywhere between the 4th and the 88th percentiles of the random-partition distribution, depending only on the space in which the split is constructed.

### 3.3. Rejection of Tree Ensembles

Random forest and gradient boosting were less predictive than the penalized linear models and failed criteria that the linear models passed. Across repeated splits, their median Q2F1 ranged from 0.34 to 0.65 compared with 0.65 to 0.80 for ridge. Gradient boosting achieved an R^2^tr of exactly 1.000 in all 250 repeats, with an s of 0.02 to 0.03 log units, indicating complete memorization of the 34 training compounds. Random forest did not reproduce the training response exactly; its median R^2^tr was 0.87 to 0.94. The two ensembles therefore failed for different reasons: gradient boosting fits the training set without residuals, whereas random forest has an effective complexity that exceeds the information content of 34 compounds, which is also why its F statistic fell below one. Support vector regression behaved like gradient boosting here, with a median R^2^tr of 0.95 to 0.99, and was rejected on the same grounds. Both ensembles also failed the Roy criterion, with Δr^2^m between 0.26 and 0.37 against a 0.2 threshold.

We derived the key evidence from a scaffold-out partition, in which 23 tryptamine analogs formed the training set and 23 5-methoxytryptamine analogs formed the test set (see Supplementary Table S4). Ridge was the only algorithm that showed positive R^2^te across all three affinity endpoints (0.652, 0.501, 0.649). Gradient boosting scored negative at 5-HT2A (−0.882) and was strongly negative at the selectivity endpoints (−2.230 and −2.562), performing worse than simply predicting the training mean. Although lasso was the best model during random resampling at 5-HT2B, it failed here (−1.497 and −0.878). This is because L1 regularization focuses on a small set of simplexes linked to the training scaffold and offers limited transferability, whereas ridge spreads weights among many correlated simplexes, enabling better transfer.

### 3.4. Contribution of Individual Labeling Schemes

Models were retrained separately for each labeling scheme (Table 4). Topological representations alone carried little information, with median Q2F1 between 0.10 and 0.17 across all three receptors, as expected, because the scaffold was constant across the series and element- and topology-labeled simplexes varied little. The physico-chemical schemes carried almost all of the signal. Refractivity alone outperformed the full descriptor set at 5-HT2A (0.825 vs. 0.715) while retaining a similar number of variables, and partial charge was the strongest single scheme at 5-HT2C. Removing partial charge lowered performance at every endpoint, so the schemes are complementary rather than redundant.

### 3.5. Stability of Simplex Selection

Across 50 repeats, lasso selected 169 distinct simplexes at 5-HT2A and 200 at 5-HT2C. None was selected in more than 80% of repeats, while 12 and 11 exceeded 40%, respectively. Coefficient magnitudes therefore cannot be interpreted from a single split, and quoting a single regression equation would misrepresent the dataset’s stability. In contrast, the coefficient signs were entirely consistent: every descriptor above 40% carried the same sign in every repeat in which it was selected. Interpretation is accordingly restricted to the direction of effect for simplexes appearing in at least 40% of repeats (Supplementary Table S6).

Of the 12 stable simplexes at 5-HT2A and the 11 at 5-HT2C, only two were shared. Three charge-labeled simplexes were stable across all three receptors and were positively charged, consistent with a common 5-HT2 recognition motif; the remainder were subtype-specific. Charge-labeled simplexes dominated the stable set, with 11 out of 12 at 5-HT2A, even though refractivity is the stronger scheme in the ablation. Individual charge simplexes appear to be sharper predictors, whereas refractivity simplexes act collectively, a pattern that ridge exploits and L1 selection does not.

### 3.6. Fragment Contribution Maps

We computed atomic contributions using the fragment-removal method of Polishchuk and colleagues [30,31], in which an atom’s contribution is the difference between the prediction for the complete molecule and the prediction after removing that atom; we obtained the latter by recomputing simplex descriptors without any simplex containing that atom. For each compound, we used only models that excluded it, yielding a median of 15 independent estimates per atom, so no contribution came from a model that saw the compound during training. This protocol is stricter than interpreting a single model fitted to all data.

Atomic contribution profiles were much less correlated across receptors than whole-molecule affinities. Across the 1692 atoms, the correlations were 0.639 for 2A/2B, 0.723 for 2B/2C, and 0.290 for 2A/2C compared with whole-molecule pKi correlations of 0.834, 0.923, and 0.766, respectively. At the whole-molecule level, 5-HT2A and 5-HT2C shared 59% of the variance, whereas at the atomic contribution level, they shared 8%. Section 3.13 repeats this analysis under a partition scheme that covered all 46 compounds and reports both sets of values.

**Figure 3:**
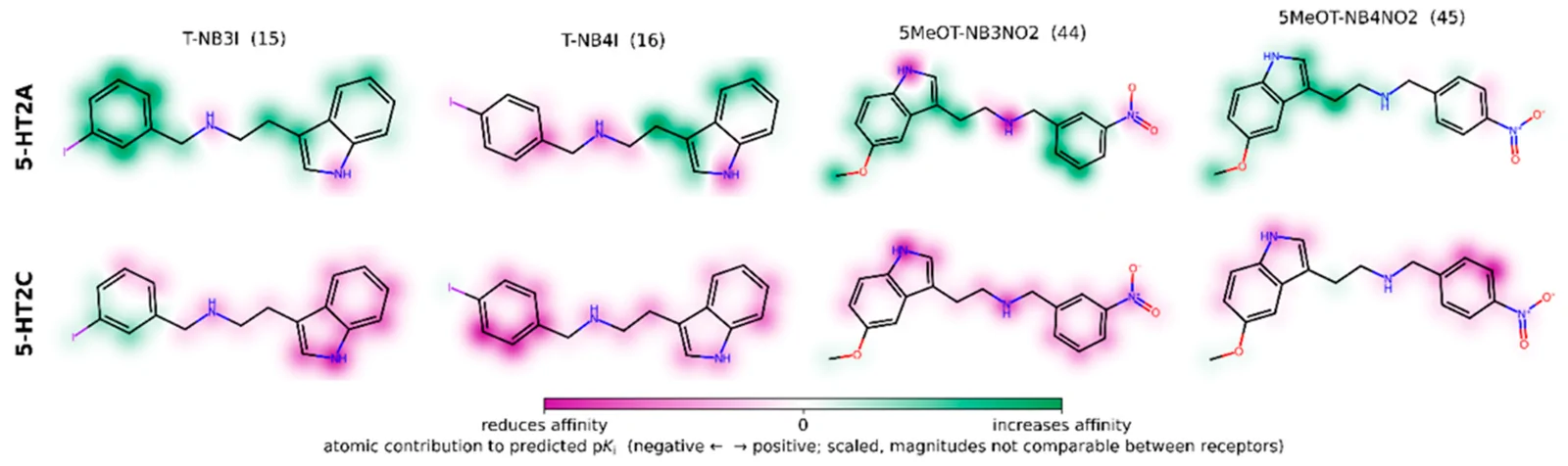
Atomic contributions to predicted pKi at 5-HT2A (**top**) and 5-HT2C (**bottom**) for four representative compounds by fragment removal with the ridge ensemble. Green raises affinity; magenta lowers it. Absolute magnitudes are not interpretable (see Section 4.4).

Figure 3 shows how individual atoms contribute to the predicted pKi at 5-HT2A (top row) and 5-HT2C (bottom row) for four representative compounds, obtained by fragment removal using the ridge ensemble. Green marks atoms that increase affinity, and magenta those that decrease it. The indole core inverts between the two receptors, and compounds 16 and 45, both with para-substituted benzyl groups, consistently showed negative contributions from the benzyl fragment. Absolute magnitudes are not meaningful (see Section 4.4); comparisons are based on rankings and differences within a receptor. The maps also make the position effect explicit. Relative to the mean contribution of the ortho and meta positions, para substitution on the benzyl ring lowered the total fragment contribution by 3.20 units at 5-HT2A, 1.80 at 5-HT2B, and 2.22 at 5-HT2C, with the largest para penalty at 5-HT2A. Within the para series, methyl is the only substituent that raises the contribution at 5-HT2A; every other substituent lowers it.

### 3.7. Limits of the Applicability Domain

We probed the domain with two deliberately demanding partitions. In the scaffold-out partition, the k-nearest-neighbor criterion flagged 83% to 100% of test compounds as outside the domain, yet PLS and ridge still predicted 5-HT2A reasonably well (R^2^te 0.715 and 0.652), so the criterion was conservative when it was not needed.

The position-out design was then run three times, withholding the ortho, meta, and para blocks in turn (Table 5). The outcome was ordered but not symmetric. With the simplex dictionary, all fifteen models trained without the para block, across five algorithms and three receptors, fell below the null model that predicts the training mean, with a median Q2F1 between −0.35 and −1.54. None of the fifteen models trained without the meta block did so, with a median Q2F1 between +0.27 and +0.47. The ortho block was intermediate in Q2F1, but its absolute error (0.42 to 0.52 log units) matched that of ordinary random partitions (0.30 to 0.42), and the negative values came from lasso and ridge rather than from all five algorithms.

The three blocks were not equally displaced from the training mean, so we also refer to each as the null model (Table 6). Withholding the meta block shifted the test mean by +1.08 log units at 5-HT2A, and withholding the para block shifted it by −1.15. Thus, the trivial predictor faces an almost identical task in both cases, with null RMSEs of 1.20 and 1.24, respectively. The models beat it on the meta block (ratio 0.86) and lost to it on the para block (ratio 1.16). The same contrast held at 5-HT2C (0.84 vs. 1.18) and 5-HT2B (0.73 vs. 1.59). Extrapolation to an unseen substitution position is therefore not, by itself, what defeated the models.

Using the PaDEL dictionary, the order was maintained at 5-HT2A and 5-HT2B, where all five models again underperformed relative to the null on the para block. However, at 5-HT2C, the order was not maintained: this para block was the best predicted among the three, and the PaDEL models at that endpoint were also the weakest in the set. Overall, PaDEL transferred less effectively to each withheld block compared to the simplex dictionary, with a ratio greater than 1.0 in seven out of nine cases, aligning with the findings in Section 3.11.

Across all three partitions and both dictionaries, the k-nearest-neighbor criterion flagged no test compounds as lying outside the domain in seven out of the nine combinations, and flagged two out of fourteen compounds in the remaining two. Mean distance ratios ranged from 0.37 to 0.81, all below the acceptance threshold of 1.0. The criterion was therefore silent on the block that is predicted well and on the block that was not predicted at all, and carried no information about which of the three could be trusted (Figure 2D and Supplementary Tables S7 and S12).

This dissociation is a result, not a defect, of the criterion. Geometric distance in simplex space does not separate para from ortho and meta sufficiently to cross the threshold, yet the response still differs. It is the model-level counterpart to the non-additivity concentrated at the para position in these data, where deviations from additivity reached 0.88 log units at 5-HT2A against an experimental noise level near 0.23.

### 3.8. Selectivity Endpoints

Neither endpoint yielded an acceptable model under design B. For ΔpKi(2A–2C), the best was ridge, with a median Q2F1 of 0.469, a CCC of 0.629, and only 40% of splits above 0.5. For ΔpKi(2A–2B), the best was elastic net at 0.536, with a CCC of 0.725. Beyond the low medians, both failed the Golbraikh–Tropsha criteria: k’ fell between 0.36 and 0.83 rather than within 0.85 to 1.15, and (r^2^ − r0^2^)/r^2^ reached 0.21 to 0.27 against a threshold of 0.1. They are therefore reported as negative results rather than as weak models.

Their failure is consistent with the position-out experiment. The selectivity signal is concentrated at the para position, where the representation loses predictive ability. Design B forces every test set to include all three positions. Under design A, the same endpoint appeared acceptable, with a median Q2F1 of 0.704 for ridge, showing how a split design can produce an apparently successful selectivity model and why the design was fixed before fitting any model.

### 3.9. Second Descriptor Dictionary (PaDEL): Rationale and Protocol

The principal claim of the preceding sections is that 5-HT2A and 5-HT2C show strong correlation in whole-molecule affinity but diverge in how their fragments contribute. Because that conclusion rested on a single fragment-based descriptor system, one could argue that the divergence reflects the representation rather than the receptors. We therefore introduced a second, structurally unrelated dictionary as a control.

We computed PaDEL-2D descriptors for the same 46 compounds, yielding 1444 variables after removing those that could not be computed. Unlike simplexes, which are sparse counts of tetratomic fragments, PaDEL descriptors are continuous variables that describe the whole molecule: constitutional indices, topological and autocorrelation descriptors, electrotopological states, BCUT eigenvalues, and calculated physicochemical properties.

Because the claim that the two dictionaries are unrelated is central to the argument, redundancy between them was measured rather than assumed (Table 7). No variables were shared. In the raw dictionaries, the median highest correlation between a simplex descriptor and its closest PaDEL descriptor was 0.89, and 47% of simplexes had a counterpart above 0.9. This figure must be read against the redundancy within each dictionary, which is higher: 93% of simplexes had a counterpart above 0.9 within the simplex dictionary itself, and 91% of PaDEL descriptors had a counterpart above 0.9 within PaDEL. Near-duplication is a property of the 46 molecules described by more than a thousand variables, and it was weaker between the two dictionaries than within either. After training-set filtering, which is what the models actually see, 15% to 25% of the retained simplexes had a PaDEL counterpart above 0.9, and the median highest correlation fell to 0.79 to 0.81. Among the descriptors that carry the interpretation, those selected by lasso in at least 40% of repeats, no cross-dictionary pair exceeded 0.9 at any receptor; the largest single value was 0.85, and the median highest correlation was 0.61 to 0.68.

The protocol was otherwise identical: design B stratification, the same 50 splits from the same random seed, and the same eight algorithms, metrics, and validation tests. Because stratification depends only on scaffold and ring position, not on descriptors, the splits were numerically identical, enabling paired split-by-split testing rather than comparing two independent distributions. One change was unavoidable: the rule requiring at least four non-zero values applies to fragment counts but not to continuous descriptors. For PaDEL, the filter removed constant variables, prescreened to the 300 most correlated with the response, and pruned greedily above |r| = 0.95. This left a median of 127 to 165 descriptors at the affinity endpoints compared with 94 to 112 for simplexes.

### 3.10. Model Performance with the Second Dictionary

We trained the same eight algorithms on the PaDEL matrix using the same 50 partitions, with hyperparameters selected via identical inner cross-validation, so any differences between the two analyses are attributable to the descriptors. The number of variables surviving training-set filtering was comparable, with medians of 127 to 165 versus 94 to 112, so differences in model dimensionality did not confound the comparison. Table 8 presents the median statistics for the four linear models at the three affinity endpoints, in the same format as Table 1; Supplementary Table S8 provides all eight algorithms and five endpoints, including interquartile ranges.

The PaDEL models met the Golbraikh–Tropsha conditions and the Q2F1 and R^2^te thresholds across all three affinity endpoints, but were less consistent with the Roy criterion compared to the simplex models. The median Δr^2^m surpassed the 0.2 threshold for ridge at all receptors (0.204, 0.211, and 0.233), for elastic net at 5-HT2B and 5-HT2C (0.212 and 0.228), and for lasso at 5-HT2C (0.222). Only PLS stayed below the threshold throughout (0.160, 0.182, and 0.188), alongside lasso and elastic net at 5-HT2A (0.130 and 0.145). The corresponding simplex values ranged from 0.126 to 0.187 at each endpoint. When selecting a single PaDEL model for comparison, PLS was the only one meeting all acceptance criteria, with the ridge model at 5-HT2C noted for comparison with that caveat.

Out-of-fold predictions from twelve repeats of stratified fourfold cross-validation yielded R^2^ values of 0.781, 0.756, and 0.726, with RMSE of 0.37, 0.30, and 0.32 log units at 5-HT2A, 5-HT2B, and 5-HT2C (Supplementary Figure S1) compared with 0.782, 0.724, and 0.819 for the simplex models. The agreement between the observed and predicted values is comparable to that in Figure 2 and shows the same structure, with no systematic departure by scaffold or substitution position. Response permutation yielded cR^2^p values between 0.63 and 0.90, with the empirical *p*-value at its lower bound for all three receptors (Table 2 and Supplementary Table S9).

**Figure 4:**
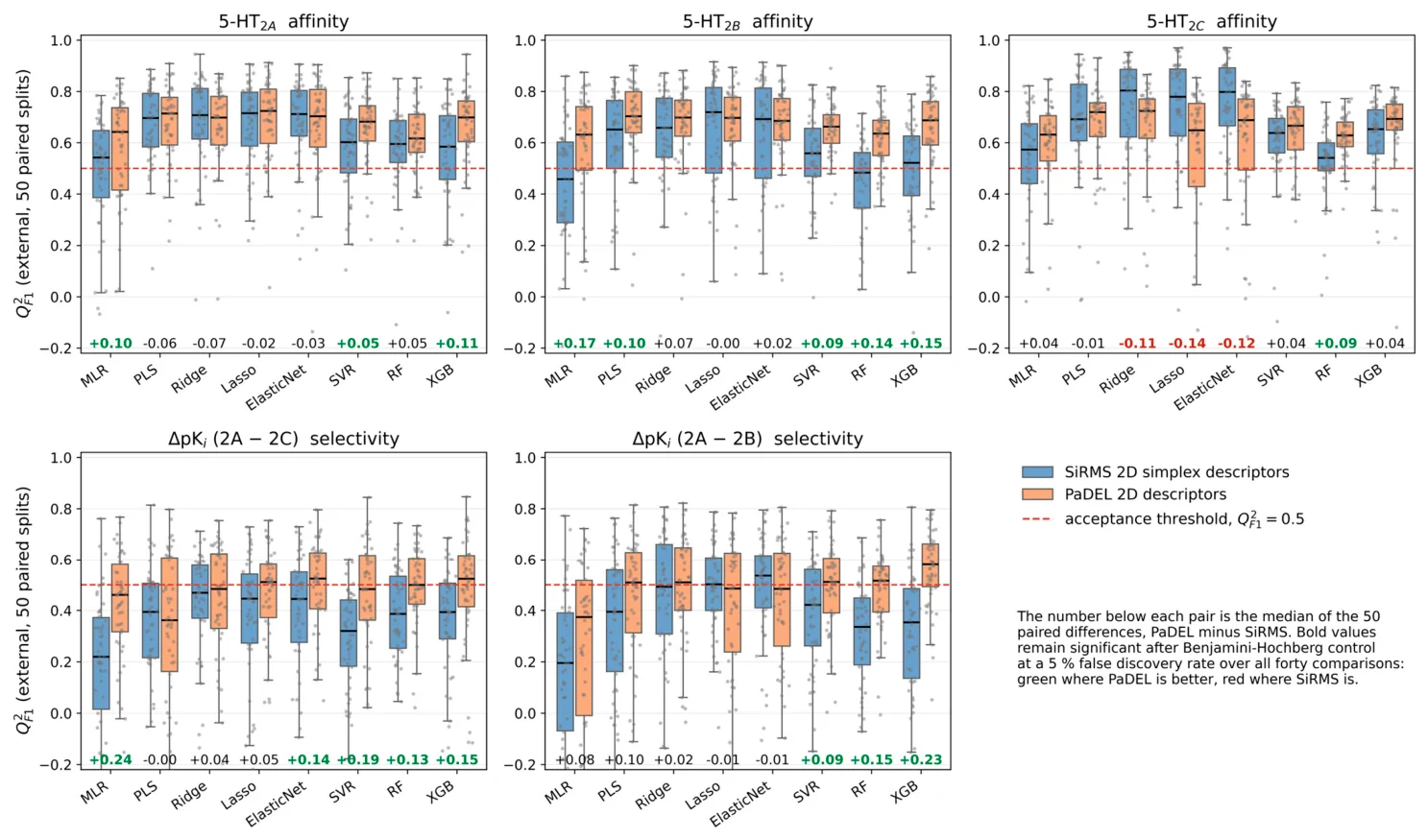
The two descriptor dictionaries across the same 50 splits, eight algorithms, and five endpoints. Blue, SiRMS; orange, PaDEL; dashed line, threshold 0.5. Numbers are medians of the 50 paired differences (PaDEL minus SiRMS), with bold values indicating significance after Benjamini–Hochberg control at 5% FDR across all forty comparisons: green favoring PaDEL, red favoring SiRMS. Full statistics in Table S10.

Figure 4 and Supplementary Table S10 present the paired comparison. For the linear models at the affinity endpoints, the two dictionaries were indistinguishable at 5-HT2A and 5-HT2B (Wilcoxon *p* between 0.14 and 0.92), as expected for two adequate representations of the same structure–activity relationship. The exception was 5-HT2C, where the simplex models performed better: ridge 0.803 against 0.724 and lasso 0.779 against 0.649, winning 66% and 76% of paired splits, respectively.

After evaluating forty comparisons, the advantage of the simplex dictionary at 5-HT2C remained significant after controlling for false discovery in all three penalized models (lasso q = 0.001, elastic net q = 0.009, ridge q = 0.024). However, only the lasso model (*p* = 0.011) remained significant under the more stringent Holm correction. The improvements of PaDEL over the simplex dictionary for tree ensembles were robust against both corrections. When applying the corrected resampled *t*-test, which accounts for overlap among the fifty training sets, none of the forty differences were statistically significant, consistent with expectations at a test-to-training ratio of 12:34. Consequently, the comparison is reported as an effect-size statement: the two dictionaries are equivalent at 5-HT2A and 5-HT2B; the simplex dictionary outperformed at 5-HT2C by a median of 0.11 to 0.14 Q2F1 units; and the tree ensembles outperformed PaDEL by 0.04 to 0.23 units. Importantly, none of these conclusions depend on the *p*-values provided.

Tree ensembles consistently behaved in the opposite manner. Gradient boosting increased from 0.585 to 0.699 at 5-HT2A and from 0.354 to 0.580 at ΔpKi(2A–2B), winning 74% and 90% of paired splits, respectively, while random forest showed improvement at every endpoint. The probable reason is that simplex descriptors are sparse counts with many zeros, making axis-aligned splits inefficient, unlike PaDEL descriptors, which are continuous. This did not change the decision to exclude the ensembles, as they still failed the Roy criterion with Δr^2^m between 0.25 and 0.50 and achieved an R^2^tr of exactly 1.000 with s below 0.01 log units in every repeat. However, it refines the interpretation: their poor performance in the simplex analysis results from an interaction between representation and algorithm, not solely from the training set size.

### 3.11. Transfer to a Related Scaffold

In the scaffold-out partition, PaDEL descriptors transferred more reliably than simplexes (Supplementary Figure S2 and Table S11). PLS models using PaDEL descriptors achieved positive R^2^te at all five endpoints: 0.648, 0.596, and 0.670 at the three receptors and 0.527 and 0.258 at the two difference endpoints, whereas the simplex models were negative at several. The asymmetry is structural: the 5-methoxytryptamine series contains simplexes absent from the tryptamine training set, so a fragment-count representation must extrapolate to descriptor values it has never seen, whereas whole-molecule descriptors such as calculated logP, molecular weight, or topological indices change smoothly as a methoxy group is added. Where the objective is transfer across a scaffold change, whole-molecule descriptors are the safer choice; where it is resolution within a fixed scaffold, simplex models are better at 5-HT2C and comparable elsewhere.

### 3.12. The Para Position Fails with Both Dictionaries

The position-out partitions were run with PaDEL descriptors under the same protocol, and the para block reproduced the simplex result at 5-HT2A and 5-HT2B: R^2^te between −7.5 and −13.2 and Q2F1 between −0.97 and +0.20, indicating predictions no better than the training mean and usually worse. The k-nearest-neighbor criterion again admitted all 14 para-substituted compounds, with a mean distance ratio between 0.37 and 0.61, well below the 1.0 threshold, and flagged none of them (Table 5 and Supplementary Table S12). Supplementary Figure S1D shows this directly for the PaDEL models and is the counterpart of Figure 2D: the para-substituted compounds carry the largest standardized residuals of the set while sitting at distances well inside the acceptance threshold, so the two quantities that a distance-based domain criterion assumes to be related are, in these data, unrelated.

This control matters for Section 3.7. The failure at the para position and the inability of a distance-based domain criterion to anticipate it are properties of the data, not of the simplex representation. Two dictionaries that shared no variables, when applied to the same compounds under the same protocol, failed at the same point and were equally silent.

### 3.13. Fragment Contributions Converge Between Dictionaries

The decisive experiment applied the fragment-removal interpretation to both dictionaries under a single protocol. Because PaDEL descriptors require chemically valid molecules, we applied the interpretation at the fragment level rather than the atomic level. We built three truncations per compound by removing the benzyl-ring substituent, the indole 5-methoxy group, and the entire N-benzyl group, yielding 108 truncated structures. We recomputed descriptors for each truncated molecule independently in both dictionaries and used only models in which the parent compound was in the test partition. Table 9 reports the mean contributions per fragment class, computed separately for the two dictionaries.

**Figure 5:**
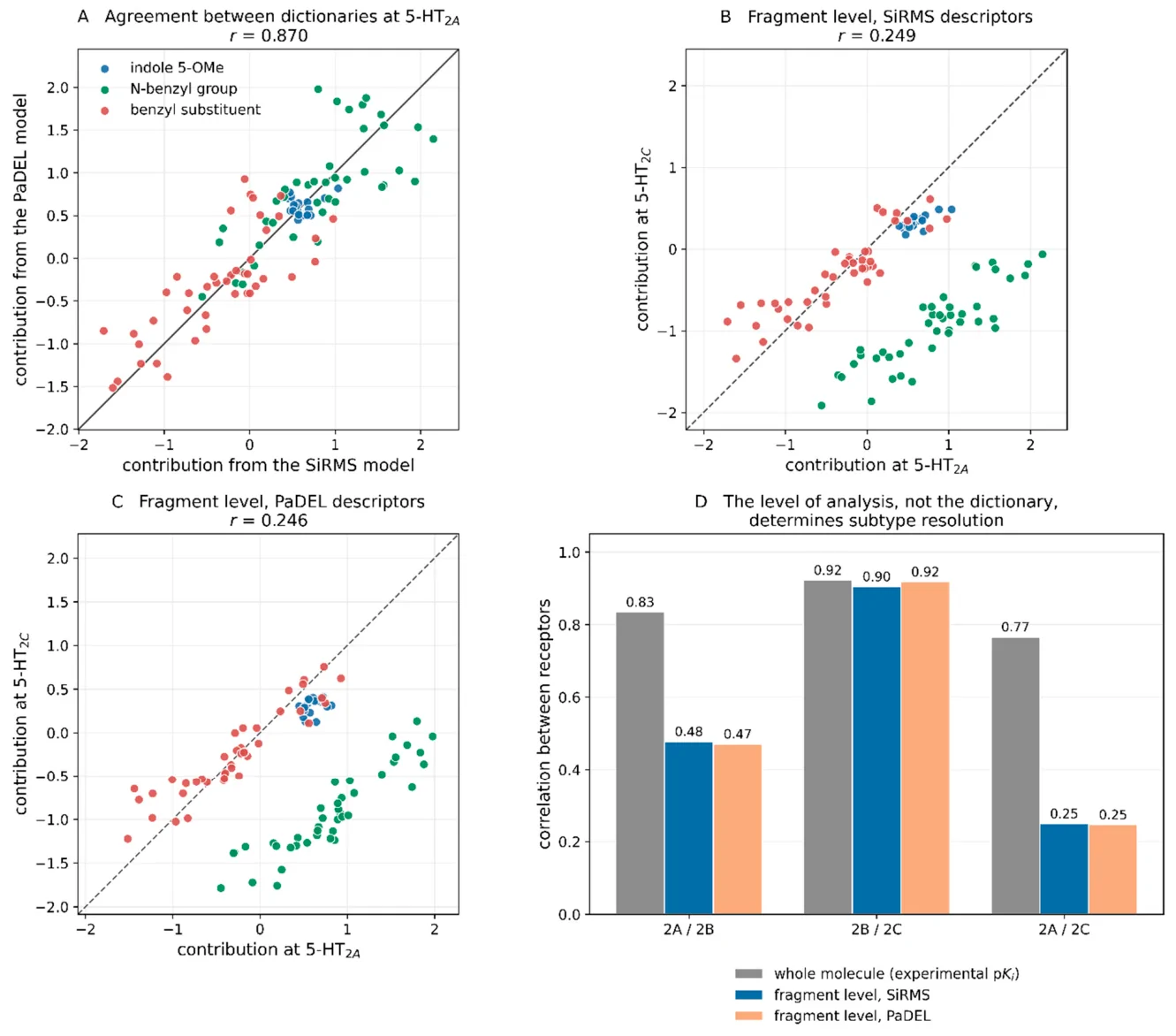
Fragment contributions from the two dictionaries. (**A**) Simplex versus PaDEL contributions at 5-HT2A; solid line, identity. (**B**,**C**) 5-HT2A versus 5-HT2C contributions for each dictionary; dashed line, identity. Colors indicate removed fragments. (**D**) Between-receptor correlation at two levels of description, whole-molecule pKi and fragment contributions.

Two key findings emerged. First, the two dictionaries showed strong agreement on each fragment’s contribution, with correlation coefficients of 0.870, 0.792, and 0.906 for 5-HT2A, 5-HT2B, and 5-HT2C, respectively (Figure 5A). Additionally, the average contributions per fragment class differed by no more than 0.06 log units. This suggests that the results are driven primarily by the data rather than by either descriptor system.

Second, contrary to our expectations, the whole-molecule dictionary reproduced the subtype divergence almost exactly. At the fragment level, the 2A/2C correlation was 0.249 with simplexes and 0.246 with PaDEL compared with 0.766 for the experimental whole-molecule pKi values (Figure 5B–D). The 2B/2C correlation remained high in both cases (0.905 and 0.918), and the 2A/2B correlation was intermediate (0.478 and 0.471). What separates the subtypes is therefore the level at which the question is asked, not the fragment nature of the descriptors: aggregating over the whole molecule conceals divergence present in the parts, and this holds whether the model is built from fragment counts or whole-molecule indices.

The overall correlation analysis should be broken down because a correlation calculated across mixed fragment classes depends not only on the location of these classes, but also on how receptors rank compounds within each class. When examined individually, 5-HT2A and 5-HT2C receptors showed high agreement: their contributions correlated at 0.870 for the N-benzyl group, 0.890 for the benzyl-ring substituent, and 0.742 for the indole 5-methoxy group. These two higher values are reflected in the entire-molecule dictionary, which showed correlations of 0.911 and 0.926. The key difference between the receptors lies in the position of one class rather than the ranking within any class. The N-benzyl group contributed +0.82 log units at 5-HT2A and −0.95 at 5-HT2C, a difference of 1.77, while the benzyl substituents differed by −0.05 and the 5-methoxy group by +0.27. Removing the N-benzyl class increased the correlation between 5-HT2A and 5-HT2C from 0.249 to 0.931 with simplex descriptors, and from 0.246 to 0.942 with PaDEL descriptors. Thus, the divergence between the two receptors is localized to a single group that reaches the extended binding pocket, which is also where their structural differences are most evident. The rest of the molecule was interpreted almost identically by both models.

Under the stratification of Section 2.3, the two parent amines and the two unsubstituted benzyl analogs do not participate in a test partition. Consequently, 105 out of the 108 truncated structures and 42 out of the 46 compounds received estimates. The three missing truncations belonged to the two unsubstituted benzyl compounds, which served as the reference members for the N-benzyl fragment series. To address this, we repeated the analysis using a cross-validation scheme in which we held out each compound twelve times, ensuring all 46 compounds and 108 truncations were evaluated (see Table 10). The overall conclusion remained the same. The fragment-level correlation between 5-HT2A and 5-HT2C was 0.242 with the simplex dictionary and 0.285 with PaDEL compared to 0.249 and 0.246 in the original scheme. The correlation between 5-HT2B and 5-HT2C remained high at 0.906 and 0.890. At the atomic level, the 5-HT2A/5-HT2C correlation increased from 0.290 to 0.381 when including the four compounds, though it was still significantly lower than the whole-molecule correlation. The N-benzyl group exhibited opposite signs across the two receptors: +0.841 at 5-HT2A versus −0.903 at 5-HT2C with simplexes, and +0.743 versus −0.919 with PaDEL.

A correlation declining from 0.766 for whole molecules to 0.249 for fragments might, in theory, reflect uneven reliability in the datasets rather than differences in the data itself, since a single contribution incurs model error twice. However, this was not the case. Each contribution was averaged over 12 to 50 models, with its parent compound held out, yielding a reliability of 0.979 to 0.995 at each receptor and under both partition schemes. The reliability of the experimental pKi values, based on replicate standard deviations across three determinations, ranged from 0.978 to 0.987. When both were disattenuated, the comparison remained consistent: the 5-HT2A/5-HT2C correlation was 0.251 at the fragment level versus 0.779 for whole molecules, and 0.244 against 0.779 under cross-validation, with atomic values of 0.305 and 0.403, respectively. An internal control confirmed this: the 5-HT2B/5-HT2C correlation, derived from the same contributions and models, measured 0.912 after correction compared to 0.942 for whole molecules. The attenuation needs to explain that the 5-HT2A/5-HT2C gap would also have to lower this pair, which it does not.

The fragment classes separated cleanly and consistently across both dictionaries. The N-benzyl group accounted for most of the subtype discrimination: removing it lowered the predicted 5-HT2A affinity by 0.82 log units on average and raised the predicted 5-HT2C affinity by 0.95, so the two effects act in opposite directions at the two receptors. The indole 5-methoxy group increased affinity across all three subtypes, most at 5-HT2A (0.61) and least at 5-HT2C (0.33). Benzyl-ring substituents had near-zero mean effects but strong positional dependence: +0.20 at meta, −0.27 at ortho, and −1.01 at para at 5-HT2A, with a 2A–2C difference of −0.27 at para against +0.06 and +0.07 at ortho and meta, reproducing the positional effect of Section 3.6 by an independent route.

### 3.14. Stability of Descriptor Selection with the Second Dictionary

Lasso selected between 135 and 137 distinct PaDEL descriptors across 50 repeats. Of these, 14 to 16 descriptors exceeded a 40% selection frequency, while 2 to 5 surpassed 80%. This indicates slightly more stability than in simplexes, where no descriptor reached 80%, reflecting a smaller and less redundant descriptor space. Sign consistency was nearly perfect. The topological charge index JGI2 was the most consistently selected descriptor across all three receptors, appearing in 100%, 94%, and 82% of repeats with a negative sign in each case. Additionally, the autocorrelation descriptor ATSC5p and the electrotopological descriptor MDEC-11 were frequently shared. As with simplexes, receptor overlap was limited: only two descriptors stable at 5-HT2A were also stable at 5-HT2C (see Supplementary Table S14).

## 4. Discussion

### 4.1. Additivity, Fragment-Level Divergence and the Limits of the Domain

The original study analyzed these data with a Free–Wilson model [17,18] and concluded that benzyl fragments behave similarly across all three subtypes, making subtype selectivity within this scaffold difficult. This conclusion follows from the analysis rather than from the chemistry. The Free–Wilson model is additive by construction and assumes no interaction between fragments. Yet these data departed from additivity, with root mean square deviations of 0.34 and 0.52 log units at 5-HT2A and 5-HT2B against an experimental noise level near 0.23, and individual deviations reaching 0.88 log units at para-nitro substitution. Kramer, Fuchs and Liedl [19] showed that non-additivity of this magnitude is common and undermines methods such as Free–Wilson analysis and matched molecular pair analysis, which assume substituent effects are independent of context. They called the phenomenon additivity cliffs by analogy with activity cliffs. Later analyses of public and in-house data [20] confirmed that non-additivity above about 0.5 log units is typical in congeneric series. A model that cannot represent interaction will report parallel fragment effects whether or not they exist.

The fragment contribution maps shifted the analysis to a different level. 5-HT2A and 5-HT2C showed strong correlation in whole-molecule affinity (r = 0.766) but weak correlation in atomic contributions from the simplex models (r = 0.290), indicating that the two subtypes differ more in where the signal resides on the molecule than in how they respond overall. The same pattern emerged in variable selection, where only about two out of a dozen stable simplexes were shared between the two receptors. Polishchuk and colleagues introduced fragment-removal interpretation to recover structural insight from models built on descriptors that cannot be read directly [30,31,43]; the present work extends that approach from ranking fragments within one endpoint to comparing fragment profiles across related endpoints.

These differences are mirrored in receptor architecture. Work on 5-HT2A selectivity identified the extended binding pocket, into which the N-benzyl group of 25CN-NBOH projects in the Gq-coupled cryo-EM structure, as the principal region of divergence between 5-HT2A and 5-HT2C. Recent structure-guided design has exploited positions within that pocket to achieve high subtype selectivity on this same scaffold [15,16]. Our maps place the largest 2A/2C differences on the benzyl fragment that reaches this region, most strongly at the para position, which sits deepest within it. This is not evidence of a binding mode, but it is consistent with the fragment-level differences reflecting receptor features rather than dataset noise.

The para position warrants attention because three independent lines of evidence converge there. Additivity testing on the raw data showed the largest deviations at para-halogen and para-nitro substitution. The contribution maps showed the largest positional penalty at para, most strongly at 5-HT2A. The position-out partition, in which models trained on ortho- and meta-substituted compounds predicted all 14 para-substituted analogs, failed outright, with median Q2F1 ranging from −0.35 to −1.54 across the three receptors. A para substituent is therefore not simply a larger substituent; it shifts the series onto a different structure–activity relationship rather than along the existing one.

By withholding one substitution position at a time, we can distinguish between two explanations that the para partition alone cannot clarify. The first suggests that extrapolation to unseen positions fails, implying a general statement about congeneric series rather than this specific chemotype. The second indicates that the para block encodes a relationship not captured by the other two positions. The data support the second explanation. The meta block is displaced from the training average by a similar magnitude as the para block but in the opposite direction, meaning that a model with no transferable knowledge would fail on both. In reality, every model outperformed the trivial predictor on the meta block but underperformed on the para block across all three receptors with the simplex dictionary, and on 5-HT2A and 5-HT2B with the whole-molecule dictionary. Conversely, the ortho block, which was close to the training mean, was predicted with an error comparable to random partitions (see Table 5 and Table 6). This reflects the model-level equivalent of the non-additivity observed directly in the raw data.

Two caveats accompany this result. Leave-class-out partitions tend to yield a conservative estimate of future performance, often more so than the optimistic bias of random selection [47]. Therefore, the statistics from all three block-out partitions should be viewed as a lower bound. The comparison between blocks remains valid, as all three were created using the same protocol, with the same level of pessimism. Additionally, at a displaced test block, R^2^ and Q2F1 reflect both the block’s position and the model’s accuracy; thus, each block can also be seen as the mean predictor’s error. The ratio of these two metrics provides insights into the model’s performance [36].

We avoid a space-covering design because it requires a different focus. Kennard–Stone and similar methods aim to make the test set representative of the training set, primarily assessing how well a model interpolates within known data. However, we were concerned with how the model performed on compounds outside the training set, which is why we used partitions that excluded an entire scaffold or substitution site. When applied to our data, the space-covering approach yielded a better estimate with the fragment-based dictionary but a less stable one with the whole-molecule dictionary; specifically, one partition dropped below 96% of the results from random partitions at one end. Including this alongside the structured partitions provides two benefits: it sets an upper bound on the range covered by the structured partitions at the lower end, and highlights the instability of a single split, showing why relying on just one partition is not reliable at this sample size.

The experiments reveal a limitation of the applicability domain. The distance-based criterion was overly cautious when unnecessary, flagging a scaffold change that ridge could handle, and silent where a warning was needed, missing the para compounds where all models failed. It added no value in any of the three positional partitions, either flagging no compounds or only two out of fourteen, regardless of whether the models succeeded or failed. The mean distance ratios ranged from 0.37 to 0.81, below the 1.0 threshold (see Table 5 and Supplementary Tables S7 and S12). Domain criteria based on geometric proximity in descriptor space assume that response discontinuities align with descriptor-space discontinuities. This assumption breaks down when small structural changes cause non-additive response changes, such as at activity or additivity cliffs [19,48]. A criterion that remains silent both when predictions are reliable and when they are not cannot assess prediction trustworthiness. Moreover, two dictionaries sharing no variables behaved identically in this regard, indicating that the behavior relates to distance in descriptor space rather than the specific representations, as Section 3.12 confirms.

A second issue concerns model complexity relative to data size. With 34 training compounds, gradient boosting fit the training data exactly in all 250 repeats and then failed to generalize to a related scaffold, sometimes performing worse than predicting the training mean, whereas penalized linear models with 15 to 21 effective parameters both predicted and transferred better. This aligns with Marchese Robinson and colleagues [49], who found no systematic accuracy advantage for random forest over regularized linear models on benchmark QSAR data, at much greater interpretive cost. We would recommend the scaffold-out partition as a routine diagnostic in small-set QSAR, since it separated algorithms more clearly than repeated random resampling.

A related question is whether models built from a pool of more than a thousand candidate descriptors on 34 training compounds are assembled from variables that correlate with the response by chance, a risk that increases with the size of the pool and decreases with the size of the sample [37]. Two results bear on this question. Reducing the pool twelvefold left the distance between internal fit and external predictivity unchanged, at 0.18 to 0.28 throughout (Table 3), as expected if the pool is used to find signal rather than fit noise. Response permutation on three partitions per endpoint placed the permuted models at zero external predictivity in every case, while some of them reached an internal R^2^ of 0.99 (Table 2). Neither result shows that the 46 compounds suffice to build a general model of this chemotype, and we do not claim that they do. They show that the statistics reported here are not a product of the search.

Two of the earlier results would still be considered provisional if based solely on the simplex models. Subtype divergence at the fragment level and the limitations of the tree ensembles both stemmed from a single descriptor system and cannot be attributed to the receptors or sample size without evidence to the contrary. Consequently, we reran the entire analysis using a second, structurally different dictionary for the same partitions. This section discusses the comparison.

### 4.2. Comparison of the Two Descriptor Dictionaries

The simplex models leave two questions open: whether the divergence between 5-HT2A and 5-HT2C at the fragment level belongs to the receptors or to the representation, and whether the weakness of the tree ensembles is intrinsic or caused by sparse fragment counts. PaDEL 2D descriptors [29] serve as a control because they share no variables with the simplex system and differ in their units of description: continuous whole-molecule indices versus counts of tetratomic fragments. Applying them to the same compounds over identical splits under an identical protocol makes the comparison paired and allows split-by-split testing rather than juxtaposing two separate analyses.

The word independent needs care here. The two dictionaries share no variables and no construction rules, but they are computed on the same 46 molecules, and any two descriptor sets computed on a small congeneric series are numerically correlated. The measured redundancy between them was lower than the redundancy within either. 47% of simplexes had a near-duplicate among the PaDEL descriptors compared with 93% within the simplex dictionary itself. Among the descriptors that survived filtering and selection, which are the ones that carry the interpretation, no cross-dictionary pair exceeded 0.9 (Table 7). The second dictionary therefore provides not statistical independence, which no descriptor set computed on the same molecules could provide, but independence of construction. Fragment counts and whole-molecule indices encode the same molecules through different primitives, and a result that survives the change of primitives is not an artifact of either.

The two dictionaries performed similarly, which was the desired and rarely tested outcome. At 5-HT2A and 5-HT2B, the linear models were statistically indistinguishable (Wilcoxon *p* between 0.14 and 0.92), and out-of-fold errors differed by less than 0.05 log units. Both dictionaries passed the same acceptance criteria and the same permutation tests. The convergence of two structurally unrelated representations provides stronger evidence for a real structure–activity relationship than either alone. One difference appeared at 5-HT2C, where simplexes outperformed PaDEL for ridge (0.803 against 0.724) and lasso (0.779 against 0.649). We report this without over-interpreting a single endpoint in a 46-compound set.

A caveat exists regarding the paired comparison. Conducting fifty repeated 75/25 partitions of 46 compounds does not constitute fifty independent experiments, as any two training sets share about two-thirds of their compounds on average. Ignoring this overlap in a paired test underestimates the variance of the difference and overstates significance [46]. Performing forty such tests worsens the issue. Correcting the raw *p*-values for multiple comparisons did not change the overall interpretation, since both the simplex advantage at 5-HT2C and the PaDEL advantage for the tree ensembles remained significant after false discovery rate correction [45]. Using the more conservative corrected resampled *t*-test, no comparisons were significant, which is expected given the test set-to-training set ratio. Consequently, we present the results as effect sizes rather than significance claims. This approach does not weaken the main argument, as the conclusions from the two dictionaries are based on their agreement: both showed similar predictive performance and reproduced each other’s fragment-level interpretation within 0.01 in receptor-pair correlations. Thus, a significance test cannot establish agreement between two representations.

The fragment-contribution results did not align with our initial hypothesis. We anticipated the divergence between subtypes to originate from the fragment-based nature of the simple representation, and the entire-molecule dictionary reflects this almost perfectly: the correlation between 2A/2C fragment contributions was 0.249 for simplexes and 0.246 for PaDEL compared to 0.766 for the actual whole-molecule pKi. Meanwhile, the 2B/2C correlation remained high in both cases (0.905 and 0.918; see Table 10). Both dictionaries also showed strong agreement on individual fragment contributions, with correlations ranging from 0.79 to 0.91, and average contributions per fragment class differing by less than 0.06 log units. Thus, the subtypes differ by the level at which the question is asked, rather than by the descriptors used.

This difference is specific, not general, and it is important to state it clearly. Within each fragment class, the two receptors agree, with correlation scores between 0.74 and 0.89 when compared to simple descriptors, meaning that neither model reorders compounds differently. The overall low correlation stems from one class behaving differently: the N-benzyl group increases the predicted 5-HT2A affinity (+0.82 log units) and decreases the 5-HT2C affinity (−0.95 log units). The other fragments contributed similarly at both receptors. Removing this class raised the correlation to 0.93. This divergence is driven by the group that interacts with the extended binding pocket, where the receptors differ in sequence, shape, and dynamics. These opposing effects cancel out when considering the entire molecule because their influences sum into a single affinity measure. An additive model that fits whole-molecule data cannot distinguish between the two, showing how aggregation masks divergence.

The divergence is also not an artifact of unequal precision between the two levels of description, the most obvious alternative explanation for a correlation dropping from 0.77 to 0.25. Contributions averaged over repeated held-out models are almost as reliable as the measured affinities themselves, and disattenuating both correlations changes neither appreciably (Supplementary Table S13b). Decisively, the 5-HT2B/5-HT2C pair was computed from the same contributions by the same models under the same noise and remained above 0.9. Whatever depresses the 5-HT2A/5-HT2C correlation at the fragment level is specific to that pair of receptors, which is the claim being made.

This revised conclusion is stronger than the original and offers a more useful critique of the Free–Wilson analysis. The problem with the original conclusion was not that Free–Wilson uses substituent indicator variables rather than fragment descriptors, since a whole-molecule model interpreted at the fragment level recovers the divergence perfectly well. The problem was that the conclusion rested on the signs of coefficients fitted separately for each receptor, an aggregate comparison, whereas the informative comparison is between profiles of fragment contributions. Selectivity depends on differences between coefficients, not on their signs, and no representation can repair a comparison made at the wrong level. The principal methodological recommendation of this work follows from this: when related endpoints are modeled, compare their interpretations, not only their predictions.

The fragment classes conveyed the same chemical message in both dictionaries. The N-benzyl group is the primary discriminator between subtypes: removing it lowered the predicted 5-HT2A affinity by 0.82 log units and raised the predicted 5-HT2C affinity by 0.95, so the two effects act in opposite directions at two receptors whose overall affinities correlate at r = 0.77. An aggregate comparison cannot capture this, and it aligns with the sequence and conformational divergence of the extended binding pocket discussed in Section 4.1 [15,16]. The indole 5-methoxy group increased affinity across all three subtypes, most at 5-HT2A, whereas benzyl substituents showed near-zero mean effects with strong positional dependence: the 2A–2C difference was −0.27 at para versus +0.06 and +0.07 at ortho and meta.

The second open question yielded a different result. Tree ensembles showed significant improvement with PaDEL descriptors, with gradient boosting increasing from 0.585 to 0.699 at 5-HT2A and from 0.354 to 0.580 at ΔpKi(2A–2B). They also went from winning 74% to 90% of paired splits (Figure 4, Supplementary Table S10). Their poor performance in the simplex analysis was partly due to the interaction between data representation and the algorithm, as axis-aligned splits are inefficient on sparse count data with many zeros. Despite this, the exclusion remains justified, since they achieved an R^2^tr of exactly 1.000 with an s below 0.01 log units in every repeat and failed the Roy criterion with a Δr^2^m between 0.25 and 0.50. In conclusion, at this sample size, ensembles tend to memorize regardless of representation, with sparse input imposing a greater penalty.

A third outcome was unexpected. In the scaffold-out partition, the dictionary ranking reversed (Supplementary Figure S2): PLS on PaDEL descriptors yielded positive R^2^te at all five endpoints, whereas the simplex models were negative at several, and the reversal was systematic across algorithms rather than confined to one. Structurally, this makes sense: the 5-methoxytryptamine series contains simplexes absent from the tryptamine training set, so a fragment-count representation must extrapolate to descriptor values it has never seen, whereas calculated logP, molecular weight, and topological indices change smoothly when a methoxy group is added. The practical lesson is that fragment-based representations resolve structure–activity relationships within a fixed scaffold, whereas whole-molecule descriptors transfer better across scaffold changes. The choice should follow the intended application rather than tradition.

The position-out experiment using the second dictionary also failed at 5-HT2A and 5-HT2B, showing R^2^te values between −7.5 and −13.2. The k-nearest-neighbor criterion again classified all 14 para-substituted compounds within the domain, with a mean distance ratio ranging from 0.37 to 0.61. Two dictionaries that shared no variables, when applied to the same compounds under the same protocol, failed similarly and were both unindicated by the same domain criterion. This dissociation between geometric distance and predictive reliability indicates a feature of the data itself rather than the simple descriptors, suggesting that domain estimation should incorporate a response-side component, such as the spread of predictions across an ensemble of models trained on different data partitions, rather than relying solely on distance.

### 4.3. Implications for This Chemotype

Two features of this series determine how it can be modeled, and both are evident in the data rather than in the modeling. The first is the concentration of non-additivity at the para position, which makes that part of the substituent space unpredictable from the rest of the series with either descriptor dictionary. Models built on these data should therefore be applied only to ortho- and meta-substituted analogs, and that restriction must be imposed on mechanistic grounds, because the distance-based domain criterion does not enforce it. The source study also reported unexpectedly high 5-HT2A potency for two ortho-substituted members of the series, for which the 25CN-NBOH structure, in which the ortho substituent is bridged to the protonated amine through S159(3.36), may offer an explanation [15,16,17]. Our results neither support nor contradict this, since ortho fragment contributions were mildly negative on average.

The second feature concerns the 5-HT2B endpoint, and one limitation constrains what we can say about it. The models are based on binding affinity, which is not efficacy. Valvular risk is driven by agonist activation of 5-HT2B rather than occupancy, so a high-affinity 5-HT2B ligand that acts as an antagonist may carry no valvular liability, while a weaker binder with high intrinsic activity may carry considerable liability. We did not model the functional data reported in the source study because 22 out of the 138 pEC50 values were censored, and censoring is associated with low activity, so removing them would bias the training set toward active compounds. Everything stated in this work about 5-HT2B should therefore be read as a statement about binding and what fragment-level analysis of binding reveals, not as a safety assessment. The practical consequence is not that the analysis is uninformative for 5-HT2B, but that it occupies an earlier position in the sequence: a fragment pattern that raises 5-HT2B affinity identifies compounds that require functional characterization and does not substitute for it.

Within those limits, the picture is unfavorable. Across the 46 compounds, the affinity difference between 5-HT2A and 5-HT2B ranged from −1.63 to +0.17 log units, so nearly every compound binds 5-HT2B at least as well as 5-HT2A, and the difference endpoint itself could not be modeled acceptably with either dictionary. Selectivity against 5-HT2B therefore lies outside the range of these data, and models built on this series cannot be used to predict it. Since 5-HT2B agonism is causally associated with valvular heart disease [9,10,11], and since the compounds in this series bind 5-HT2B with nanomolar affinity and act as agonists in calcium-mobilization assays, this is a liability that ligand-based modeling can measure on the binding side but cannot repair. 5-HT2B-sparing analogs would have to be developed experimentally rather than selected computationally.

### 4.4. Limitations and Scope

The set includes 46 compounds from a single laboratory and assay platform, with all statistics reflecting that uncertainty. Interquartile ranges are consistently reported, and no single data split is highlighted as the definitive model. Absolute fragment contributions are not interpretable because removing structural elements successively counts overlapping descriptors multiple times. Therefore, we focused only on the rankings, differences within a receptor, and correlations between receptor profiles.

Four compounds, the two parent amines and the two unsubstituted benzyl analogs, were grouped into two strata with two members each, so they did not participate in the test partition during any of the fifty repeats. Their contributions were still recovered through the repeated cross-validation scheme, where each compound was held out twelve times, leaving the fragment-level conclusions unchanged (Section 3.13). Notably, the atomic 5-HT2A/5-HT2C correlation increased significantly from 0.29 to 0.38 after including these four compounds, and both values are reported.

The discretization boundaries for the simplex labeling schemes are more general than those used in earlier studies [26,27]. This prevents direct numerical comparison with previous simplex literature but still allows for valid internal comparisons. We analyzed fragment-level results across three fragment classes rather than per atom because the whole-molecule dictionary requires chemically valid truncated structures. This approach is coarser than the atom-level maps in Section 3.6, so any agreement should be evaluated at the fragment-class level.

No compound was synthesized, no prediction was tested prospectively, and the models are not offered as tools for prospective design. The object of study was an existing set of 138 measured affinities, and the question is what an additive model does and does not recover from it. Thus, the claims made here are about that dataset and the analyses applied to it. The structured partitions serve as a surrogate for prospective performance that these data permit: a model trained without an entire scaffold, or without an entire substitution position, is asked to predict compounds that differ from its training set as a genuinely new compound would. Partitions of this kind give a pessimistic rather than an optimistic estimate of prospective accuracy [47], so what is reported is a lower bound. The principal conclusion is moreover negative: fragment effects diverge between 5-HT2A and 5-HT2C, and the models fail on para-substituted analogs without warning from the applicability domain. A negative conclusion of this kind is not made safer by synthesizing compounds; it is made safer by reproducing it under a second descriptor representation, under three independent partition schemes, and under response permutation on three partitions per endpoint, as was carried out here.

## 5. Conclusions

A reexamination of a complete set of 46 N-benzyltryptamines and N-benzyl-5-methoxytryptamines reveals that the earlier conclusion from additive analysis, that benzyl fragments behave similarly across all three 5-HT2 receptor subtypes, making selective targeting difficult, is not supported by these findings. The analysis shows that additivity does not hold in this series, with deviations reaching up to 0.88 log units compared to an experimental noise level of about 0.23. This failure was most pronounced at the para position, where the selectivity signal was strongest. The additive model, however, did not indicate this failure; instead, it redistributed the discrepancy among its coefficients, resulting in a seemingly acceptable fit.

Interpreting validated non-additive models at the structural-fragment level reveals a different picture. 5-HT2A and 5-HT2C correlated at 0.77 for whole-molecule affinity and at 0.25 for fragment contributions, with the N-benzyl group acting in opposite directions at the two receptors. This is not a property of the descriptor system: two dictionaries that share no variables, one built on local fragment counts and the other on whole-molecule indices, yielded the same figures to within 0.003. The difference has a single identifiable origin. The N-benzyl group contributes +0.82 log units at 5-HT2A and −0.95 at 5-HT2C, while every other fragment class contributes almost equally at both, so excluding the N-benzyl group alone restores a correlation of 0.93. Summing over the whole molecule cancels those opposing contributions, which is why an additive model fitted to whole-molecule affinities reports parallel fragment effects at the two receptors. Fragment contributions correlated between 0.79 and 0.91 across dictionaries, and disattenuating for estimate reliability left the comparison unchanged. The decisive factor is the level at which the comparison is made, since aggregation over the whole molecule conceals divergence visible at the parts. Fragment-level interpretation of related endpoints is therefore worth performing routinely, even when the aggregate models look uninformative.

Three practical points emerge for a series of this size. First, penalized linear models with 15 to 20 effective parameters outperformed tree ensembles in both prediction and transfer; gradient boosting fitted the training data exactly in every repeat and performed worse than the training mean in the scaffold-out test. Its performance improved markedly on continuous rather than sparse inputs, so its limitations depend on the interaction among representation, algorithm, and sample size. Second, fragment-count descriptors resolved the structure–activity relationship within a fixed scaffold but transferred poorly across scaffold changes, whereas whole-molecule descriptors transferred better at a small cost in resolution, so the choice of descriptor should follow the intended application. Third, distance-based applicability-domain criteria were unreliable exactly where they were needed: every model failed on para-substituted analogs, while both descriptor sets placed those compounds inside the domain, and withholding the ortho or the meta block instead did not produce the same failure. Geometric proximity in descriptor space does not anticipate response discontinuities caused by non-additivity, so domain estimation should include a response-side component, such as the dispersion of an ensemble trained on different partitions.

Two chemotype limits are outlined below. Future models based on these data should be applied only to ortho- and meta-substituted analogs, since the present data and methods cannot reliably predict the para region. Selectivity against 5-HT2B is unlikely to be achievable within this series, since the affinity difference between 5-HT2A and 5-HT2B ranges from −1.63 to +0.17 log units, with most compounds favoring the antitarget, and the corresponding difference endpoint cannot be modeled acceptably. Because valvular risk depends on agonist efficacy at 5-HT2B rather than affinity alone, ligand-based modeling can flag this property on the binding side but cannot resolve it. The series would need experimental extension to identify 5-HT2B-sparing analogs rather than screening for them computationally.

## Figures and Tables

**Table 1:** Median performance of the four linear models over 50 stratified 75/25 splits under design B.

Endpoint/Model	*p*	R^2^tr	F	s	Q^2^F1	IQR of Q^2^F1	R^2^te	r^2^m	Δr^2^m	RMSE	MAE	CCC
5-HT2A PLS	3	0.932	137.4	0.225	0.696	0.58–0.79	0.687	0.596	0.182	0.422	0.344	0.837
5-HT2A Ridge	18	0.948	18.732	0.262	0.708	0.61–0.81	0.707	0.613	0.179	0.405	0.319	0.847
5-HT2A Lasso	18	0.964	19.601	0.259	0.716	0.59–0.80	0.713	0.624	0.186	0.422	0.335	0.846
5-HT2A ElasticNet	28	0.952	3.723	0.441	0.712	0.63–0.80	0.711	0.621	0.175	0.405	0.319	0.852
5-HT2B PLS	3	0.924	121.4	0.177	0.652	0.50–0.77	0.637	0.527	0.187	0.353	0.265	0.802
5-HT2B Ridge	18	0.941	14.480	0.211	0.658	0.54–0.77	0.617	0.502	0.181	0.350	0.261	0.792
5-HT2B Lasso	18	0.959	23.524	0.187	0.719	0.48–0.81	0.711	0.583	0.166	0.332	0.251	0.842
5-HT2B ElasticNet	23	0.958	13.370	0.223	0.692	0.46–0.81	0.659	0.542	0.182	0.342	0.252	0.811
5-HT2C PLS	3	0.946	176.3	0.149	0.692	0.61–0.83	0.683	0.616	0.164	0.303	0.234	0.841
5-HT2C Ridge	21	0.986	35.814	0.128	0.803	0.62–0.89	0.798	0.713	0.131	0.247	0.200	0.878
5-HT2C Lasso	20	0.985	35.992	0.131	0.779	0.63–0.89	0.775	0.675	0.140	0.253	0.200	0.878
5-HT2C ElasticNet	26	0.985	12.005	0.199	0.799	0.67–0.89	0.791	0.686	0.126	0.251	0.197	0.886

*p*, effective number of parameters: trace of the hat matrix for ridge, number of non-zero coefficients for lasso and elastic net, number of latent variables for PLS. F and s are computed against that value. IQR, interquartile range of Q^2^F1. r^2^m, Roy average; Δr^2^m, the corresponding difference. Statistics for all eight algorithms and all five endpoints are given in Supplementary Table S2.

**Table 2:** Response permutation on three partitions per endpoint.

Dictionary/ Endpoint	cR^2^p q25	cR^2^p Median	cR^2^p q75	Real Q^2^F1 Range	Max Scrambled Q^2^F1
SiRMS 5-HT2A	0.908	0.895	0.900	0.66 to 0.80	0.47
SiRMS 5-HT2B	0.863	0.741	0.897	0.78 to 0.78	0.44
SiRMS 5-HT2C	0.896	0.909	0.876	0.71 to 0.88	0.59
PaDEL 5-HT2A	0.864	0.825	0.844	0.60 to 0.84	0.51
PaDEL 5-HT2B	0.873	0.822	0.851	0.65 to 0.76	0.63
PaDEL 5-HT2C	0.836	0.852	0.875	0.64 to 0.78	0.52

cR^2^p and the real Q^2^F1 are given for ridge; the median partition is the one reported in the original submission. The last column is the highest permuted Q^2^F1 over all three algorithms on the two additional partitions. Full results by algorithm and partition are in Supplementary Tables S3 and S9.

**Table 3:** Sensitivity of the ridge models to the size of the prescreened descriptor pool.

Dictionary/ Endpoint	Desc. at 25	Desc. at 300	Q^2^F1 25	Q^2^F1 50	Q^2^F1 100	Q^2^F1 300	Gap 25	Gap 300
SiRMS 5-HT2A	16	105	0.625	0.651	0.707	0.708	0.18	0.24
SiRMS 5-HT2B	16	97	0.428	0.485	0.557	0.658	0.28	0.28
SiRMS 5-HT2C	16	94	0.572	0.651	0.691	0.803	0.27	0.18
PaDEL 5-HT2A	17	134	0.645	0.672	0.690	0.698	0.19	0.24
PaDEL 5-HT2B	19	165	0.631	0.668	0.675	0.698	0.21	0.22
PaDEL 5-HT2C	20	127	0.630	0.690	0.708	0.724	0.25	0.20

Medians over the same 50 splits of design B. “desc.” is the median number of descriptors surviving filtering at that pool size, and “gap” is the difference between R^2^tr and Q^2^F1 at that pool size. Lasso follows the same pattern and is given in Supplementary Table S16.

**Table 4:** Median external predictivity of models built on individual atom-labeling schemes.

Labelling Scheme	Raw	Filtered	5-HT2A	5-HT2B	5-HT2C
Element	287	39	0.096	0.168	0.107
Topology	11	5	0.168	0.175	0.169
Partial charge	648	135	0.709	0.664	0.754
Lipophilicity	318	104	0.380	0.116	0.374
Refractivity	311	110	0.825	0.695	0.694
H-bond	89	12	0.136	0.111	0.075
Element + topology	298	41	0.096	0.170	0.109
All physico-chemical	1366	113	0.715	0.641	0.834
All six schemes	1664	105	0.715	0.668	0.803
All minus charge	1016	72	0.497	0.406	0.655

Values are median Q^2^F1 for ridge models over 50 splits under design B. “Raw” is the number of descriptors produced by the scheme and “Filtered” the median number surviving training-set filtering, both for the 5-HT2A endpoint. Full statistics are in Supplementary Table S5.

**Table 5:** Position-out partitions: withholding the ortho, the meta, and the para block in turn.

Dictionary/ Endpoint	Ortho Q^2^F1	Meta Q^2^F1	Para Q^2^F1	Ortho RMSE	Meta RMSE	Para RMSE	Below Null
SiRMS 5-HT2A	−0.05	+0.27	−0.35	0.52	1.03	1.44	3/0/5
SiRMS 5-HT2B	+0.19	+0.47	−1.54	0.43	0.73	1.16	2/0/5
SiRMS 5-HT2C	+0.04	+0.30	−0.39	0.42	0.76	1.06	2/0/5
PaDEL 5-HT2A	−0.29	−0.19	−0.63	0.58	1.31	1.59	4/3/5
PaDEL 5-HT2B	+0.35	+0.12	−0.30	0.38	0.95	0.83	1/1/5
PaDEL 5-HT2C	−0.59	−0.17	+0.04	0.54	0.99	0.88	3/5/1

Medians over five algorithms (PLS, ridge, lasso, random forest, gradient boosting); RMSE in log units. The last column shows how many of the five models fell below the training-mean null predictor for the ortho, meta, and para blocks, respectively. Full results by algorithm are in Supplementary Tables S7 and S12.

**Table 6:** Each withheld block referred to the null model that predicts the training mean.

Endpoint/Block	Training Mean	Block Mean	Offset	Null RMSE	SiRMS RMSE	Ratio
5-HT2A ortho	6.72	6.87	+0.16	0.51	0.52	1.03
5-HT2A meta	6.44	7.51	+1.08	1.20	1.03	0.86
5-HT2A para	7.12	5.96	−1.15	1.24	1.44	1.16
5-HT2B ortho	7.24	7.06	−0.17	0.47	0.43	0.90
5-HT2B meta	6.93	7.76	+0.82	1.01	0.73	0.73
5-HT2B para	7.39	6.71	−0.68	0.73	1.16	1.59
5-HT2C ortho	6.76	6.61	−0.16	0.42	0.42	0.98
5-HT2C meta	6.48	7.27	+0.79	0.91	0.76	0.84
5-HT2C para	6.98	6.12	−0.86	0.90	1.06	1.18

All values in log units. The null RMSE is the error of a predictor that assigns the training-set mean to every test compound. A ratio below 1 means that the model carries information beyond that mean.

**Table 7:** Numerical redundancy within and between the two descriptor dictionaries.

Comparison	n SiRMS	n PaDEL	Median Highest |r|	% Above 0.9	% Above 0.95
Raw dictionaries	1595	986	0.89	47	37
Within SiRMS	1595	—	1.00	93	84
Within PaDEL	—	986	0.99	91	79
After filtering, 5-HT2A	106	143	0.81	25	10
After filtering, 5-HT2B	97	167	0.81	15	5
After filtering, 5-HT2C	97	143	0.79	16	4
Stable selections, 5-HT2A	12	16	0.68	0	0
Stable selections, 5-HT2B	8	14	0.62	0	0
Stable selections, 5-HT2C	11	15	0.61	0	0

For each descriptor, we take the highest absolute correlation to any descriptor in the comparison set across the 46 compounds; the table reports the median of those values and the proportion exceeding 0.9 and 0.95. Stable selections are descriptors chosen by lasso in at least 40% of the 50 repeats. Descriptors with zero variance across the 46 compounds are excluded, hence 1595 and 986. Full values are in Supplementary Table S15.

**Table 8:** Median performance of the PaDEL-based linear models over the same 50 splits used for the simplex models.

Endpoint/Model	*p*	R^2^tr	F	s	Q^2^F1	IQR of Q^2^F1	R^2^te	r^2^m	Δr^2^m	RMSE	MAE	CCC
5-HT2A PLS	4	0.908	87.934	0.261	0.714	0.59–0.78	0.695	0.583	0.160	0.437	0.351	0.826
5-HT2A Ridge	14	0.935	19.537	0.277	0.698	0.59–0.78	0.677	0.556	0.204	0.442	0.347	0.825
5-HT2A Lasso	18	0.956	20.064	0.249	0.724	0.60–0.81	0.710	0.599	0.130	0.427	0.345	0.842
5-HT2A ElasticNet	32	0.956	2.099	0.541	0.704	0.58–0.81	0.696	0.595	0.145	0.431	0.349	0.835
5-HT2B PLS	3	0.900	78.261	0.204	0.704	0.64–0.80	0.682	0.574	0.182	0.328	0.253	0.837
5-HT2B Ridge	13	0.919	18.015	0.220	0.698	0.63–0.77	0.679	0.534	0.211	0.341	0.262	0.819
5-HT2B Lasso	18	0.948	16.999	0.200	0.697	0.61–0.78	0.686	0.538	0.192	0.349	0.265	0.809
5-HT2B ElasticNet	29	0.950	2.482	0.373	0.685	0.61–0.77	0.676	0.535	0.212	0.344	0.265	0.807
5-HT2C PLS	3	0.905	93.029	0.203	0.719	0.62–0.76	0.699	0.577	0.188	0.333	0.268	0.830
5-HT2C Ridge	13	0.926	20.444	0.216	0.724	0.62–0.77	0.715	0.510	0.233	0.342	0.267	0.818
5-HT2C Lasso	18	0.958	20.636	0.182	0.649	0.43–0.75	0.634	0.529	0.222	0.352	0.279	0.805
5-HT2C ElasticNet	27	0.956	4.795	0.317	0.688	0.49–0.77	0.682	0.545	0.228	0.347	0.282	0.815

Columns are defined as in Table 1. Because the partitions are numerically identical to those used for the simplex models, values in this table are directly comparable row by row with those of Table 1. Statistics for all eight algorithms and all five endpoints are given in Supplementary Table S8.

**Table 9:** Mean fragment contributions obtained independently from the two descriptor dictionaries.

Fragment	SiRMS 2A	SiRMS 2B	SiRMS 2C	PaDEL 2A	PaDEL 2B	PaDEL 2C
N-benzyl	0.820	−0.216	−0.950	0.819	−0.241	−0.926
Substituent	−0.359	0.047	−0.310	−0.299	0.031	−0.253
5-OMe	0.606	0.436	0.332	0.604	0.402	0.306

Values are in log units. A positive value indicates that removing the fragment lowers the predicted affinity, so the fragment contributes favorably at that receptor. Contributions were obtained by fragment removal with the ridge ensemble, using only models in which the compound appeared in the test partition. Absolute magnitudes are not meaningful; see Section 4.4. Supplementary Table S13 reports contributions with standard deviations and the corresponding values under the cross-validation scheme.

**Table 10:** Contribution profiles under the two partition schemes.

Level/Dictionary/Scheme	*n*	2A/2B	2B/2C	2A/2C
Fragment, SiRMS, 50 repeated splits	105	0.478	0.905	0.249
Fragment, SiRMS, repeated 4-fold CV	108	0.483	0.906	0.242
Fragment, PaDEL, 50 repeated splits	105	0.471	0.918	0.246
Fragment, PaDEL, repeated 4-fold CV	108	0.555	0.890	0.285
Atom, SiRMS, 50 repeated splits	1692	0.639	0.723	0.290
Atom, SiRMS, repeated 4-fold CV	1822	0.597	0.804	0.381
Whole-molecule experimental pKi	46	0.834	0.923	0.766

Pearson correlations between contribution profiles for each receptor pair. The last row shows the correlation of measured pKi values for level comparison. Disattenuated values are in Supplementary Table S13b.

## Data Availability

The data presented in this study are available in the article and in the Supplementary Materials. The complete analysis code, the descriptor matrices, and all derived result files are openly available in Zenodo 1.1 at https://doi.org/10.5281/zenodo.22961405, with the version accompanying this revision archived separately. The experimental affinity values reanalyzed here were reported in [17] and are reproduced with attribution; readers using those values should cite the original publication.

## Supplementary Materials

The following supporting information can be downloaded at: https://www.mdpi.com/article/10.3390/molecules31203764/s1, Table S1: Consistency checks applied to the programmatically generated structures; Table S2: Median statistics for all eight algorithms and all five endpoints, simplex descriptors; Table S3: Response permutation on three partitions for each affinity endpoint, simplex descriptors; Table S4: Scaffold-out partition: training on the tryptamine series, testing on the 5-methoxytryptamine series, simplex descriptors; Table S5: Full statistics for models built on individual atom-labelling schemes, simplex descriptors; Table S6: Simplexes selected by lasso in at least 40% of the 50 repeats, with the direction of their coefficient; Table S7: Position-out partitions: withholding the ortho, the meta and the para block in turn, simplex descriptors; Table S8: Median statistics for all eight algorithms and all five endpoints, PaDEL descriptors; Table S9: Response permutation on three partitions for each affinity endpoint, PaDEL descriptors; Table S10: Split-by-split comparison of the two descriptor dictionaries over identical partitions, with Benjamini–Hochberg and Holm adjustment and the corrected resampled *t* test; Table S11: Scaffold-out partition: training on the tryptamine series, testing on the 5-methoxytryptamine series, PaDEL descriptors; Table S12: Position-out partitions: withholding the ortho, the meta and the para block in turn, PaDEL descriptors; Table S13: Mean fragment contributions with standard deviations by descriptor dictionary, receptor and partition scheme; Table S13b: Correlation of contribution profiles between receptors by level, dictionary and partition scheme with disattenuation; Table S14: PaDEL descriptors selected by lasso in at least 40% of the 50 repeats, with the direction of their coefficient; Table S15: Numerical redundancy within and between the two descriptor dictionaries; Table S16: Model performance as a function of the number of descriptors surviving univariate prescreening; Figure S1: Observed versus out-of-fold predicted pKi from the PaDEL-based ridge models with the applicability domain; Figure S2: Transfer from the tryptamine series to the 5-methoxytryptamine series for both descriptor dictionaries. Table S17: A Kennard–Stone deterministic space-covering partition evaluated against the distribution of the fifty stratified partitions for both dictionaries.

## Abbreviations

The following abbreviations are used in this manuscript:

5-HT	5-hydroxytryptamine (serotonin)
AD	applicability domain
CCC	concordance correlation coefficient
CV	cross-validation
FDR	false discovery rate
HRMS	high-resolution mass spectrometry
InChI	International Chemical Identifier
IQR	interquartile range
MAE	mean absolute error
MLR	multiple linear regression
NBOH	N-(2-hydroxybenzyl)
NBOMe	N-(2-methoxybenzyl)
PaDEL	software for the calculation of molecular descriptors and fingerprints
PLS	partial least squares
QSAR	quantitative structure–activity relationship
RF	random forest
RMSE	root mean square error
SiRMS	simplex representation of molecular structure
SMARTS	SMILES arbitrary target specification
SMILES	simplified molecular input line entry system
SPCI	structural and physico-chemical interpretation
SVR	support vector regression
XGB	extreme gradient boosting
