# Supplementary material for: QSAR Modeling and Fragment Contribution Analysis of N-Benzyltryptamines at Human 5-HT2 Receptor Subtypes
Source: Molecules. 2026 Oct 20;31(20):3764. doi: 10.3390/molecules31203764 (PMC13707879; doi:10.3390/molecules31203764)
Supplement: Supplementary file 1 [file molecules-31-03764-s001.zip › molecules-4582823-supplementary.pdf]

Supplementary Material

QSAR modelling and fragment contribution analysis of N-benzyltryptamines at human 5-HT2 receptor subtypes

Tables S1 to S7 relate to the simplex models of Part I of the Results; Tables S8 to S14 and Figures S1 and S2 relate to the second descriptor dictionary of Part II. Tables are numbered in the order in which they are first cited. Because the two analyses use numerically identical partitions, tables with the same structure can be read row by row against each other.

S1. Dataset and structure validation

The complete dataset is provided as the machine-readable file dataset.xlsx, containing for each of the 46 compounds the compound number, code, series, indole substitution, benzyl position and substituent, canonical SMILES of the free base, molecular formula, molecular weight, InChIKey, pKi with standard deviation and Ki in nM at all three receptors, and the three derived difference endpoints as live formulas. Structures were assembled programmatically from scaffold and substituent tokens; the scripts paper_data.py and build_smiles.py reproduce the generation and all validation checks.

Table S1. Consistency checks applied to the programmatically generated structures.

Check	What it tests	Result
C1	RDKit parses every SMILES	46/46
C2	Molecular formula from SMILES equals the formula derived independently from the compound name	46/46
C3	ortho, meta or para pattern verified by graph distance within the benzyl ring	44/44 substituted analogues
C4	Exact [M+H]+ mass against the HRMS calcd values printed in the source paper	5/5 within 0.0015 Da
C5	All InChIKeys unique	46 distinct
C6	Methoxy on indole C5 rather than C4, C6 or C7	all 5-MeO analogues match
C7	Internal consistency of transcribed pKi against Ki in nM	138/138 within 0.02 log units

C4 is an external check, since the reference values originate from the source publication rather than from this work. C6 compares compound 23 with an independently written reference structure and matches all remaining 5-methoxy analogues against a SMARTS pattern.

S2. Complete performance statistics, simplex descriptors

Table S2. Median statistics for all eight algorithms and all five endpoints, simplex descriptors.

Endpoint	Model	p	R^2tr	F	s	Q^2F1	IQR	R^2te	r^2m	Δr^2m	k	k'	$(r^2-r0^2)/r^2$	RMSE	MAE	CCC	f>0.5
5-HT2A	MLR	4	0.816	32.143	0.370	0.542	0.39-0.65	0.538	0.434	0.207	0.994	0.998	0.030	0.555	0.427	0.744	0.56
5-HT2A	PLS	3	0.932	137.4	0.225	0.696	0.58-0.79	0.687	0.596	0.182	0.998	0.997	0.029	0.422	0.344	0.837	0.86
5-HT2A	Ridge	18	0.948	18.732	0.262	0.708	0.61-0.81	0.707	0.613	0.179	1.001	0.994	0.014	0.405	0.319	0.847	0.82

Endpoint	Model	p	R^2tr	F	s	Q^2F1	IQR	R^2te	r^2m	Δr^2m	k	k'	$(r^2-r0^2)/r^2$	RMSE	MAE	CCC	f>0.5
5-HT2A	Lasso	18	0.964	19.601	0.259	0.716	0.59-0.80	0.713	0.624	0.186	1.007	0.990	0.023	0.422	0.335	0.846	0.82
5-HT2A	ElasticNet	28	0.952	3.723	0.441	0.712	0.63-0.80	0.711	0.621	0.175	1.007	0.991	0.025	0.405	0.319	0.852	0.86
5-HT2A	SVR	105	0.986	0.621	0.552	0.602	0.48-0.69	0.595	0.407	0.282	1.001	0.992	0.038	0.480	0.373	0.759	0.74
5-HT2A	RF	105	0.901	0.090	1.469	0.595	0.52-0.69	0.588	0.404	0.303	1.000	0.993	0.032	0.510	0.371	0.742	0.78
5-HT2A	XGB	105	1.000	196.8	0.032	0.585	0.46-0.71	0.575	0.449	0.197	0.994	1.001	0.020	0.504	0.357	0.761	0.64
5-HT2B	MLR	4	0.800	29.023	0.293	0.458	0.29-0.60	0.402	0.363	0.179	1.000	0.996	0.071	0.461	0.345	0.699	0.46
5-HT2B	PLS	3	0.924	121.4	0.177	0.652	0.50-0.76	0.637	0.527	0.187	1.002	0.996	0.049	0.353	0.265	0.802	0.74
5-HT2B	Ridge	18	0.941	14.480	0.211	0.658	0.54-0.77	0.617	0.502	0.181	1.001	0.996	0.033	0.350	0.261	0.792	0.80
5-HT2B	Lasso	18	0.959	23.524	0.187	0.719	0.48-0.82	0.711	0.583	0.166	1.002	0.996	0.033	0.332	0.251	0.842	0.74
5-HT2B	ElasticNet	23	0.958	13.370	0.223	0.692	0.46-0.81	0.659	0.542	0.182	1.000	0.997	0.029	0.342	0.252	0.811	0.70
5-HT2B	SVR	97	0.946	0.190	0.785	0.559	0.47-0.66	0.545	0.310	0.332	0.999	0.998	0.058	0.414	0.313	0.669	0.66
5-HT2B	RF	97	0.871	0.070	1.287	0.484	0.35-0.56	0.453	0.214	0.369	0.997	0.999	0.059	0.474	0.351	0.606	0.44
5-HT2B	XGB	97	1.000	132.8	0.031	0.522	0.39-0.63	0.503	0.326	0.260	0.999	0.998	0.045	0.439	0.321	0.691	0.52
5-HT2C	MLR	4	0.834	36.556	0.265	0.573	0.44-0.67	0.564	0.423	0.203	1.000	0.995	0.028	0.394	0.308	0.738	0.64
5-HT2C	PLS	3	0.946	176.3	0.149	0.692	0.61-0.83	0.683	0.616	0.164	1.003	0.996	0.026	0.303	0.234	0.841	0.86
5-HT2C	Ridge	21	0.986	35.814	0.128	0.803	0.62-0.89	0.798	0.713	0.131	1.006	0.994	0.029	0.247	0.200	0.878	0.88
5-HT2C	Lasso	20	0.985	35.992	0.131	0.779	0.63-0.89	0.775	0.675	0.140	1.007	0.993	0.016	0.253	0.200	0.878	0.86
5-HT2C	ElasticNet	26	0.985	12.005	0.199	0.799	0.67-0.89	0.791	0.686	0.126	1.007	0.992	0.016	0.251	0.197	0.886	0.90
5-HT2C	SVR	94	0.993	0.995	0.288	0.638	0.56-0.69	0.612	0.384	0.332	1.000	0.997	0.066	0.360	0.281	0.744	0.86
5-HT2C	RF	94	0.890	0.084	1.185	0.541	0.49-0.60	0.534	0.295	0.370	0.992	1.005	0.046	0.420	0.319	0.649	0.68
5-HT2C	XGB	94	1.000	271.3	0.023	0.653	0.56-0.73	0.636	0.442	0.267	0.998	0.998	0.033	0.365	0.289	0.761	0.82
D(2A-2C)	MLR	4	0.842	38.588	0.241	0.220	0.02-0.37	0.136	0.196	0.059	0.738	0.455	0.208	0.330	0.276	0.449	0.10
D(2A-2C)	PLS	4	0.894	69.930	0.197	0.394	0.22-0.51	0.269	0.320	0.062	0.715	0.558	0.093	0.302	0.253	0.584	0.26
D(2A-2C)	Ridge	10	0.884	16.858	0.232	0.469	0.37-0.58	0.401	0.361	0.093	0.863	0.555	0.082	0.268	0.228	0.629	0.40

Endpoint	Model	p	R^2tr	F	s	Q^2F1	IQR	R^2te	r^2m	Δr^2m	k	k'	$(r^2 - r0^2)/r^2$	RMSE	MAE	CCC	f>0.5
D(2A-2C)	Lasso	13	0.906	15.194	0.220	0.446	0.27-0.54	0.347	0.335	0.069	0.795	0.593	0.108	0.282	0.233	0.618	0.34
D(2A-2C)	ElasticNet	25	0.906	3.028	0.352	0.445	0.28-0.55	0.362	0.334	0.073	0.769	0.612	0.139	0.282	0.236	0.624	0.40
D(2A-2C)	SVR	112	0.968	0.273	0.560	0.320	0.18-0.44	0.191	0.219	0.070	0.893	0.357	0.208	0.305	0.258	0.471	0.10
D(2A-2C)	RF	112	0.909	0.090	0.973	0.387	0.25-0.53	0.297	0.291	0.064	0.989	0.438	0.124	0.289	0.247	0.491	0.30
D(2A-2C)	XGB	112	1.000	202.7	0.022	0.394	0.29-0.51	0.271	0.254	0.078	0.843	0.505	0.112	0.298	0.247	0.532	0.28
D(2A-2B)	MLR	4	0.758	23.234	0.244	0.196	-0.07-0.39	0.156	0.179	0.100	0.850	0.706	0.272	0.344	0.294	0.488	0.12
D(2A-2B)	PLS	4	0.878	54.491	0.179	0.395	0.16-0.56	0.353	0.330	0.107	0.922	0.760	0.079	0.305	0.253	0.635	0.36
D(2A-2B)	Ridge	14	0.925	14.450	0.177	0.493	0.31-0.66	0.457	0.378	0.166	0.953	0.801	0.053	0.270	0.225	0.683	0.48
D(2A-2B)	Lasso	19	0.938	12.865	0.178	0.502	0.40-0.61	0.476	0.449	0.150	0.932	0.825	0.055	0.265	0.215	0.727	0.50
D(2A-2B)	ElasticNet	26	0.937	3.627	0.267	0.536	0.41-0.62	0.502	0.441	0.135	0.937	0.833	0.054	0.262	0.212	0.725	0.64
D(2A-2B)	SVR	105	0.954	0.204	0.567	0.422	0.26-0.56	0.349	0.224	0.215	0.945	0.739	0.073	0.297	0.248	0.527	0.34
D(2A-2B)	RF	105	0.891	0.080	0.893	0.336	0.19-0.45	0.289	0.159	0.223	0.958	0.702	0.097	0.312	0.264	0.497	0.18
D(2A-2B)	XGB	105	1.000	231.1	0.018	0.354	0.14-0.48	0.307	0.248	0.102	0.954	0.703	0.104	0.314	0.259	0.550	0.22

Medians over 50 stratified 75/25 splits under design B. p, effective number of parameters; IQR, interquartile range of Q^2F1; f>0.5, fraction of splits exceeding 0.5. Acceptance thresholds: Q^2F1 > 0.5, R^2te > 0.6, r^2m > 0.5, Δr^2m < 0.2, 0.85 < k, k' < 1.15, $(r^2 - r0^2)/r^2 < 0.1$. Note that gradient boosting reaches R^2tr of exactly 1.000 at every endpoint.

S3. Y-randomisation, simplex descriptors

Table S3. Response permutation on three partitions for each affinity endpoint, simplex descriptors.

Endpoint	Partition	Model	R2 real	Q2 real	R2 scr, median	Q2 scr, median	Q2 scr, max	cR2p	p
5-HT2A	lower quartile	PLS	0.874	0.717	0.419	-0.555	0.464	0.631	0.020
5-HT2A	lower quartile	Ridge	0.962	0.661	0.021	-0.012	0.354	0.908	0.020
5-HT2A	lower quartile	Lasso	0.968	0.634	0.000	0.000	0.306	0.897	0.020
5-HT2A	median	PLS	0.895	0.533	0.408	-0.498	0.338	0.651	0.020
5-HT2A	median	Ridge	0.958	0.701	0.027	-0.008	0.197	0.895	0.020
5-HT2A	median	Lasso	0.955	0.614	0.000	0.000	0.393	0.889	0.020
5-HT2A	upper quartile	PLS	0.875	0.780	0.430	-0.410	0.472	0.610	0.032
5-HT2A	upper quartile	Ridge	0.998	0.804	0.116	-0.009	0.404	0.900	0.032
5-HT2A	upper quartile	Lasso	0.992	0.833	0.014	0.000	0.334	0.939	0.032
5-HT2B	lower quartile	PLS	0.835	0.711	0.490	-0.519	0.440	0.556	0.032
5-HT2B	lower quartile	Ridge	0.913	0.782	0.016	-0.010	0.309	0.863	0.032
5-HT2B	lower quartile	Lasso	0.937	0.570	0.014	0.000	0.268	0.847	0.032

Endpoint	Partition	Model	R2 real	Q2 real	R2 scr, median	Q2 scr, median	Q2 scr, max	cR2p	p
5-HT2B	median	PLS	0.819	0.673	0.426	-0.238	0.584	0.570	0.020
5-HT2B	median	Ridge	0.786	0.577	0.017	-0.008	0.196	0.741	0.020
5-HT2B	median	Lasso	0.951	0.808	0.000	0.000	0.311	0.877	0.020
5-HT2B	upper quartile	PLS	0.915	0.658	0.425	-0.453	0.170	0.653	0.032
5-HT2B	upper quartile	Ridge	0.957	0.775	0.032	-0.017	0.302	0.897	0.032
5-HT2B	upper quartile	Lasso	0.972	0.789	0.000	0.000	0.244	0.925	0.032
5-HT2C	lower quartile	PLS	0.876	0.642	0.396	-0.299	0.591	0.650	0.032
5-HT2C	lower quartile	Ridge	0.969	0.714	0.020	-0.007	0.124	0.896	0.032
5-HT2C	lower quartile	Lasso	0.952	0.617	0.000	0.000	0.375	0.914	0.032
5-HT2C	median	PLS	0.847	0.861	0.457	-0.396	0.624	0.575	0.020
5-HT2C	median	Ridge	0.995	0.808	0.021	-0.007	0.214	0.909	0.020
5-HT2C	median	Lasso	0.976	0.750	0.000	0.000	0.238	0.904	0.020
5-HT2C	upper quartile	PLS	0.863	0.710	0.427	-0.541	0.464	0.617	0.032
5-HT2C	upper quartile	Ridge	0.952	0.884	0.016	-0.003	0.374	0.876	0.032
5-HT2C	upper quartile	Lasso	0.969	0.890	0.000	0.000	0.238	0.923	0.032

The three partitions are those whose ridge Q2F1 lies closest to the lower quartile, the median and the upper quartile of the distribution over the 50 repeats; the median partition is the one reported in the original submission. Fifty permutations on the median partition and thirty on the other two, so the empirical p value is bounded below by 0.020 and 0.032 respectively. Under a permuted response the lasso shrinks to the intercept, so its scrambled statistics are zero by construction; the informative case is PLS, which retains a fixed number of latent variables and reaches scrambled R2tr as high as 0.99 while its external Q2 stays between -0.19 and -0.56.

S4. Scaffold-out partition, simplex descriptors

Table S4. Scaffold-out partition: training on the tryptamine series, testing on the 5-methoxytryptamine series, simplex descriptors.

Endpoint	Model	R^2tr	R^2te	CCC	RMSE
5-HT2A	MLR	0.792	-0.628	0.483	1.034
5-HT2A	PLS	0.773	0.715	0.830	0.433
5-HT2A	Ridge	0.820	0.652	0.782	0.478
5-HT2A	Lasso	0.866	0.601	0.815	0.512
5-HT2A	ElasticNet	0.890	0.652	0.820	0.478
5-HT2A	SVR	0.833	-0.738	0.042	1.069
5-HT2A	RF	0.881	-0.724	0.253	1.064
5-HT2A	XGB	1.000	-0.882	0.232	1.112
5-HT2B	MLR	0.770	0.161	0.651	0.594
5-HT2B	PLS	0.917	-0.799	0.440	0.870
5-HT2B	Ridge	0.695	0.501	0.618	0.458
5-HT2B	Lasso	0.939	-1.497	0.332	1.025
5-HT2B	ElasticNet	0.923	-0.902	0.399	0.894
5-HT2B	SVR	0.964	-0.410	0.007	0.770
5-HT2B	RF	0.834	0.265	0.317	0.556
5-HT2B	XGB	1.000	0.239	0.359	0.566
5-HT2C	MLR	0.835	-2.746	0.276	1.208
5-HT2C	PLS	0.773	0.530	0.753	0.428
5-HT2C	Ridge	0.853	0.649	0.790	0.369
5-HT2C	Lasso	1.000	-0.878	0.497	0.855

Endpoint	Model	R^2tr	R^2te	CCC	RMSE
5-HT2C	ElasticNet	1.000	-0.373	0.570	0.731
5-HT2C	SVR	0.891	-0.141	0.082	0.666
5-HT2C	RF	0.852	0.395	0.453	0.485
5-HT2C	XGB	1.000	0.181	0.458	0.565
D(2A-2C)	MLR	0.693	-0.796	0.356	0.706
D(2A-2C)	PLS	0.691	0.264	0.637	0.452
D(2A-2C)	Ridge	0.708	0.203	0.575	0.470
D(2A-2C)	Lasso	0.815	-0.313	0.455	0.603
D(2A-2C)	ElasticNet	0.841	-0.122	0.527	0.558
D(2A-2C)	SVR	0.934	-0.241	0.000	0.587
D(2A-2C)	RF	0.856	-1.598	0.174	0.849
D(2A-2C)	XGB	1.000	-2.230	0.259	0.946
D(2A-2B)	MLR	0.875	-4.567	0.229	1.021
D(2A-2B)	PLS	0.969	-4.570	0.256	1.021
D(2A-2B)	Ridge	0.959	-3.475	0.286	0.915
D(2A-2B)	Lasso	0.977	-6.145	0.223	1.157
D(2A-2B)	ElasticNet	0.976	-5.481	0.239	1.102
D(2A-2B)	SVR	0.955	-0.450	0.187	0.521
D(2A-2B)	RF	0.880	-0.911	0.263	0.598
D(2A-2B)	XGB	1.000	-2.562	0.207	0.817

Single deterministic partition of 23 training and 23 test compounds. R^2te is computed against the mean of the test set and is the appropriate statistic here, because the 5-methoxy series is systematically more potent and Q^2F1 would be inflated by the offset between the two series. Negative values indicate performance worse than predicting the training mean.

S5. Descriptor block ablation

Table S5. Full statistics for models built on individual atom-labelling schemes, simplex descriptors.

Labelling scheme	Raw	n 2A	R^2tr 2A	Q^2F1 2A	q25 2A	CCC 2A	n 2B	R^2tr 2B	Q^2F1 2B	q25 2B	CCC 2B	n 2C	R^2tr 2C	Q^2F1 2C	q25 2C	CCC 2C
Element	287	39	0.290	0.096	-0.029	0.167	38	0.379	0.168	0.076	0.229	39	0.342	0.107	0.009	0.161
Topology	11	5	0.293	0.168	0.079	0.292	6	0.285	0.175	0.066	0.297	6	0.324	0.169	0.051	0.225
Partial charge	648	135	0.893	0.709	0.606	0.834	126	0.911	0.664	0.581	0.799	127	0.942	0.754	0.677	0.853
Lipophilicity	318	104	0.742	0.380	0.133	0.605	104	0.641	0.116	-0.007	0.361	104	0.760	0.374	0.128	0.567
Refractivity	311	110	0.935	0.825	0.752	0.903	111	0.899	0.695	0.643	0.804	111	0.874	0.694	0.584	0.807
H-bond	89	12	0.307	0.136	0.004	0.232	12	0.304	0.111	0.007	0.221	12	0.336	0.075	0.001	0.177
Element + topology	298	41	0.295	0.096	0.005	0.168	40	0.380	0.170	0.091	0.231	40	0.342	0.109	0.015	0.162
All physico-chemical	1366	113	0.956	0.715	0.631	0.851	108	0.937	0.641	0.499	0.800	108	0.977	0.834	0.651	0.897
All six schemes	1664	105	0.951	0.715	0.595	0.850	97	0.933	0.668	0.521	0.793	94	0.980	0.803	0.648	0.885

Labelling scheme	Raw	n 2A	R^2tr 2A	Q^2F1 2A	q25 2A	CCC 2A	n 2B	R^2tr 2B	Q^2F1 2B	q25 2B	CCC 2B	n 2C	R^2tr 2C	Q^2F1 2C	q25 2C	CCC 2C
All minus charge	1016	72	0.826	0.497	0.290	0.692	77	0.833	0.406	0.209	0.597	82	0.973	0.655	0.465	0.786

Ridge models, design B, 50 splits. Raw, number of descriptors generated by the scheme; n, median number surviving training-set filtering at that endpoint; q25, lower quartile of Q^2F1. Discretisation boundaries were −0.05, 0 and 0.05 for partial charge; −0.2, 0 and 0.2 for lipophilicity; 1.5, 2.5 and 3.5 for refractivity; hydrogen bonding was encoded as three categorical labels.

S6. Stable simplexes

Table S6. Simplexes selected by lasso in at least 40 % of the 50 repeats, with the direction of their coefficient.

Endpoint	Simplex	Scheme	Selection frequency	Sign
5-HT2A	REFRACTIVITY|A.C(-D)=D	REFRACTIVITY	0.76	+1
5-HT2A	CHARGE|A-D.B=C	CHARGE	0.66	+1
5-HT2A	CHARGE|B-B.B=D	CHARGE	0.60	+1
5-HT2A	CHARGE|B-B=C-B	CHARGE	0.60	+1
5-HT2A	CHARGE|A(-B=C)=B	CHARGE	0.58	-1
5-HT2A	CHARGE|A-A=B-C	CHARGE	0.58	+1
5-HT2A	CHARGE|A(-B)=B-C	CHARGE	0.56	-1
5-HT2A	CHARGE|A-B.C-D	CHARGE	0.52	-1
5-HT2A	CHARGE|A-A.B-D	CHARGE	0.46	+1
5-HT2A	CHARGE|A-A-D.D	CHARGE	0.46	+1
5-HT2A	CHARGE|A-B.A-B	CHARGE	0.46	-1
5-HT2A	CHARGE|A-A.B=D	CHARGE	0.42	+1
5-HT2B	CHARGE|B-B=C-B	CHARGE	0.74	+1
5-HT2B	CHARGE|A-A.D-D	CHARGE	0.66	+1
5-HT2B	CHARGE|A-A=B.B	CHARGE	0.58	+1
5-HT2B	CHARGE|A(-B)=B-D	CHARGE	0.52	-1
5-HT2B	REFRACTIVITY|A-C=C-A	REFRACTIVITY	0.50	-1
5-HT2B	elm|C(-H)(-H)-H	elm	0.48	+1
5-HT2B	CHARGE|A-A.B-D	CHARGE	0.46	+1
5-HT2B	CHARGE|A-A=B-C	CHARGE	0.42	+1
5-HT2C	CHARGE|B-B=C-B	CHARGE	0.76	+1
5-HT2C	CHARGE|A(-B)=B-D	CHARGE	0.74	-1
5-HT2C	CHARGE|A-C.C-D	CHARGE	0.58	-1
5-HT2C	CHARGE|A(-B)=B.B	CHARGE	0.52	-1
5-HT2C	CHARGE|A-C.A-C	CHARGE	0.52	-1
5-HT2C	LOGP|C-C-C=D	LOGP	0.50	+1
5-HT2C	CHARGE|A(-C-D)-D	CHARGE	0.50	-1
5-HT2C	REFRACTIVITY|A-C(-C)=D	REFRACTIVITY	0.48	-1
5-HT2C	REFRACTIVITY|A-C-C-A	REFRACTIVITY	0.44	-1
5-HT2C	CHARGE|A-A=B-C	CHARGE	0.42	+1
5-HT2C	CHARGE|A.A(-B)=B	CHARGE	0.40	-1

Sign consistency was 1.00 for every entry, that is, each descriptor carried the same sign in every repeat in which it was selected. No descriptor was selected in more than 80 % of repeats. Notation follows the SiRMS convention, letters denoting the discretised label of each vertex. Complete selection frequencies for all descriptors are in stability_pKi_5HT2A.csv, stability_pKi_5HT2B.csv and stability_pKi_5HT2C.csv.

S7. Position-out partition, simplex descriptors

Table S7. Position-out partitions: withholding the ortho, the meta and the para block in turn, simplex descriptors.

Withheld block	Endpoint	Model	R2te	Q2F1	RMSE	CCC	Outside AD	Mean AD ratio
ortho	5-HT2A	PLS	0.2	0.248	0.44	0.678	14%	0.81
ortho	5-HT2A	Ridge	-0.3	-0.191	0.56	0.610	14%	0.81
ortho	5-HT2A	Lasso	-2.4	-2.083	0.89	0.414	14%	0.81
ortho	5-HT2A	RF	0.1	0.185	0.46	0.552	14%	0.81
ortho	5-HT2A	XGB	-0.2	-0.051	0.52	0.494	14%	0.81
ortho	5-HT2B	PLS	0.2	0.315	0.39	0.638	0%	0.77
ortho	5-HT2B	Ridge	-0.4	-0.188	0.52	0.494	0%	0.77
ortho	5-HT2B	Lasso	-3.2	-2.689	0.91	0.187	0%	0.77
ortho	5-HT2B	RF	0.1	0.190	0.43	0.427	0%	0.77
ortho	5-HT2B	XGB	0.2	0.292	0.40	0.511	0%	0.77
ortho	5-HT2C	PLS	0.3	0.432	0.32	0.675	0%	0.72
ortho	5-HT2C	Ridge	-1.3	-0.982	0.60	0.333	0%	0.72
ortho	5-HT2C	Lasso	-8.2	-6.910	1.19	-0.035	0%	0.72
ortho	5-HT2C	RF	-0.1	0.042	0.41	0.331	0%	0.72
ortho	5-HT2C	XGB	0.1	0.255	0.37	0.495	0%	0.72
ortho	D(2A-2C)	PLS	0.1	0.605	0.26	0.722	0%	0.66
ortho	D(2A-2C)	Ridge	0.4	0.732	0.21	0.773	0%	0.66
ortho	D(2A-2C)	Lasso	0.3	0.722	0.22	0.660	0%	0.66
ortho	D(2A-2C)	RF	-0.2	0.496	0.29	0.472	0%	0.66
ortho	D(2A-2C)	XGB	-0.3	0.451	0.31	0.333	0%	0.66
ortho	D(2A-2B)	PLS	0.3	0.726	0.22	0.736	0%	0.57
ortho	D(2A-2B)	Ridge	0.3	0.742	0.21	0.723	0%	0.57
ortho	D(2A-2B)	Lasso	0.2	0.684	0.23	0.589	0%	0.57
ortho	D(2A-2B)	RF	-0.3	0.520	0.29	0.381	0%	0.57
ortho	D(2A-2B)	XGB	-0.3	0.513	0.29	0.351	0%	0.57
meta	5-HT2A	PLS	-2.2	0.377	0.95	0.200	0%	0.65
meta	5-HT2A	Ridge	-2.6	0.301	1.00	0.190	0%	0.65
meta	5-HT2A	Lasso	-2.8	0.262	1.03	0.098	0%	0.65
meta	5-HT2A	RF	-2.8	0.268	1.03	0.141	0%	0.65
meta	5-HT2A	XGB	-2.8	0.264	1.03	0.190	0%	0.65
meta	5-HT2B	PLS	-0.5	0.508	0.71	0.338	0%	0.42
meta	5-HT2B	Ridge	-0.6	0.476	0.73	0.351	0%	0.42
meta	5-HT2B	Lasso	-0.9	0.382	0.79	0.298	0%	0.42
meta	5-HT2B	RF	-0.8	0.419	0.77	0.248	0%	0.42
meta	5-HT2B	XGB	-0.6	0.471	0.73	0.288	0%	0.42
meta	5-HT2C	PLS	-1.9	0.276	0.78	0.148	0%	0.42
meta	5-HT2C	Ridge	-1.1	0.471	0.66	0.460	0%	0.42
meta	5-HT2C	Lasso	-1.1	0.476	0.66	0.466	0%	0.42

Withheld block	Endpoint	Model	R2te	Q2F1	RMSE	CCC	Outside AD	Mean AD ratio
meta	5-HT2C	RF	-1.8	0.299	0.76	0.163	0%	0.42
meta	5-HT2C	XGB	-1.8	0.301	0.76	0.243	0%	0.42
meta	D(2A-2C)	PLS	-0.2	0.313	0.37	0.347	0%	0.34
meta	D(2A-2C)	Ridge	-0.0	0.378	0.35	0.367	0%	0.34
meta	D(2A-2C)	Lasso	-0.2	0.286	0.38	0.257	0%	0.34
meta	D(2A-2C)	RF	-0.3	0.249	0.39	0.377	0%	0.34
meta	D(2A-2C)	XGB	-0.5	0.096	0.43	0.274	0%	0.34
meta	D(2A-2B)	PLS	-0.9	0.102	0.33	0.446	0%	0.44
meta	D(2A-2B)	Ridge	-1.2	-0.082	0.36	0.414	0%	0.44
meta	D(2A-2B)	Lasso	-0.8	0.148	0.32	0.466	0%	0.44
meta	D(2A-2B)	RF	-1.1	-0.008	0.35	0.359	0%	0.44
meta	D(2A-2B)	XGB	-1.7	-0.281	0.40	0.332	0%	0.44
para	5-HT2A	PLS	-10.1	-0.547	1.55	0.129	0%	0.57
para	5-HT2A	Ridge	-8.7	-0.346	1.44	0.119	0%	0.57
para	5-HT2A	Lasso	-7.0	-0.112	1.31	0.153	0%	0.57
para	5-HT2A	RF	-8.1	-0.266	1.40	0.097	0%	0.57
para	5-HT2A	XGB	-10.1	-0.551	1.55	0.107	0%	0.57
para	5-HT2B	PLS	-20.5	-1.588	1.17	0.154	0%	0.63
para	5-HT2B	Ridge	-20.4	-1.569	1.16	0.145	0%	0.63
para	5-HT2B	Lasso	-15.0	-0.918	1.01	0.183	0%	0.63
para	5-HT2B	RF	-14.2	-0.831	0.98	0.134	0%	0.63
para	5-HT2B	XGB	-20.1	-1.535	1.16	0.142	0%	0.63
para	5-HT2C	PLS	-15.4	-0.411	1.07	0.156	0%	0.51
para	5-HT2C	Ridge	-15.3	-0.400	1.07	0.149	0%	0.51
para	5-HT2C	Lasso	-12.6	-0.165	0.97	0.162	0%	0.51
para	5-HT2C	RF	-14.7	-0.349	1.05	0.097	0%	0.51
para	5-HT2C	XGB	-15.1	-0.385	1.06	0.141	0%	0.51
para	D(2A-2C)	PLS	-0.9	-0.114	0.48	0.239	0%	0.37
para	D(2A-2C)	Ridge	-0.9	-0.110	0.48	0.262	0%	0.37
para	D(2A-2C)	Lasso	-1.1	-0.244	0.51	0.238	0%	0.37
para	D(2A-2C)	RF	-0.6	0.060	0.44	0.393	0%	0.37
para	D(2A-2C)	XGB	-1.0	-0.138	0.48	0.387	0%	0.37
para	D(2A-2B)	PLS	-0.3	0.500	0.43	0.348	0%	0.37
para	D(2A-2B)	Ridge	-1.8	-0.100	0.64	0.206	0%	0.37
para	D(2A-2B)	Lasso	-0.6	0.365	0.48	0.333	0%	0.37
para	D(2A-2B)	RF	-1.2	0.140	0.56	0.213	0%	0.37
para	D(2A-2B)	XGB	-0.9	0.241	0.53	0.257	0%	0.37

Training set 32 compounds in each case, test set the 14 compounds of the withheld position. The para columns reproduce the partition reported in the original submission. Outside AD is the fraction of test compounds flagged by the k-nearest-neighbour criterion, and the mean AD ratio is the mean distance to the training set divided by the acceptance threshold, so values below 1 lie inside the domain. All fifteen models trained without the para block fall below a mean-predictor baseline at all three receptors, none of the fifteen trained without the meta block does, and the criterion admits essentially every compound in all three partitions.

S8. Complete performance statistics, PaDEL descriptors

Table S8. Median statistics for all eight algorithms and all five endpoints, PaDEL descriptors.

Endpoint	Model	p	R²tr	F	s	Q²F1	IQR	R²te	r²m	Δr²m	k	k'	(r²−r0²)/r²	RMSE	MAE	CCC	f>0.5
5-HT2A	MLR	4	0.839	37.669	0.345	0.642	0.42-0.74	0.635	0.485	0.166	1.001	0.995	0.040	0.476	0.400	0.789	0.64
5-HT2A	PLS	4	0.908	87.934	0.261	0.714	0.59-0.78	0.695	0.583	0.160	1.004	0.991	0.012	0.437	0.351	0.826	0.86
5-HT2A	Ridge	14	0.935	19.537	0.277	0.698	0.59-0.78	0.677	0.556	0.204	1.005	0.992	0.019	0.442	0.347	0.825	0.86
5-HT2A	Lasso	18	0.956	20.064	0.249	0.724	0.60-0.81	0.710	0.599	0.130	1.003	0.992	0.026	0.427	0.345	0.842	0.88
5-HT2A	ElasticNet	32	0.956	2.099	0.541	0.704	0.58-0.81	0.696	0.595	0.145	1.003	0.991	0.025	0.431	0.349	0.835	0.82
5-HT2A	SVR	134	0.996	1.945	0.287	0.683	0.61-0.74	0.671	0.457	0.278	1.008	0.988	0.035	0.450	0.357	0.780	0.90
5-HT2A	RF	134	0.926	0.096	1.277	0.617	0.56-0.71	0.610	0.284	0.419	1.004	0.989	0.086	0.504	0.403	0.704	0.82
5-HT2A	XGB	134	1.000	1694.4	0.010	0.699	0.60-0.76	0.689	0.443	0.275	1.005	0.992	0.027	0.457	0.351	0.797	0.88
5-HT2B	MLR	4	0.835	36.692	0.256	0.632	0.49-0.74	0.620	0.521	0.219	1.004	0.994	0.042	0.358	0.297	0.790	0.70
5-HT2B	PLS	3	0.900	78.261	0.204	0.704	0.64-0.80	0.682	0.574	0.182	1.005	0.992	0.022	0.328	0.253	0.837	0.90
5-HT2B	Ridge	13	0.919	18.015	0.220	0.698	0.63-0.77	0.679	0.534	0.211	1.005	0.993	0.038	0.341	0.262	0.819	0.86
5-HT2B	Lasso	18	0.948	16.999	0.200	0.697	0.61-0.78	0.686	0.538	0.192	1.007	0.991	0.027	0.349	0.265	0.809	0.88
5-HT2B	ElasticNet	29	0.950	2.482	0.373	0.685	0.61-0.77	0.676	0.535	0.212	1.007	0.992	0.019	0.344	0.265	0.807	0.88
5-HT2B	SVR	165	0.993	0.837	0.281	0.663	0.60-0.71	0.654	0.366	0.366	1.002	0.995	0.092	0.357	0.281	0.761	0.92
5-HT2B	RF	165	0.926	0.077	0.959	0.636	0.55-0.69	0.614	0.235	0.496	1.004	0.994	0.129	0.395	0.303	0.698	0.84
5-HT2B	XGB	165	1.000	913.1	0.009	0.688	0.59-0.76	0.654	0.434	0.253	1.003	0.995	0.047	0.366	0.268	0.784	0.90
5-HT2C	MLR	4	0.871	48.847	0.238	0.632	0.53-0.71	0.604	0.457	0.220	1.002	0.996	0.040	0.382	0.301	0.782	0.82
5-HT2C	PLS	3	0.905	93.029	0.203	0.719	0.62-0.76	0.699	0.577	0.188	1.005	0.994	0.024	0.333	0.268	0.830	0.82
5-HT2C	Ridge	13	0.926	20.444	0.216	0.724	0.62-0.77	0.715	0.510	0.233	1.006	0.991	0.050	0.342	0.267	0.818	0.84
5-HT2C	Lasso	18	0.958	20.636	0.182	0.649	0.43-0.75	0.634	0.529	0.222	1.004	0.992	0.053	0.352	0.279	0.805	0.72
5-HT2C	ElasticNet	27	0.956	4.795	0.317	0.688	0.49-0.77	0.682	0.545	0.228	1.006	0.991	0.046	0.347	0.282	0.815	0.74
5-HT2C	SVR	127	0.994	1.253	0.281	0.667	0.57-0.74	0.660	0.392	0.329	0.998	1.000	0.077	0.342	0.268	0.770	0.86
5-HT2C	RF	127	0.935	0.113	0.913	0.629	0.58-0.68	0.620	0.253	0.498	0.998	1.000	0.126	0.371	0.297	0.697	0.92

Endpoint	Model	p	R^2tr	F	s	Q^2F1	IQR	R^2te	r^2m	Δr^2m	k	k'	$(r^2-r0^2)/r^2$	RMSE	MAE	CCC	f>0.5
5-HT2C	XGB	127	1.000	1924.0	0.007	0.693	0.65-0.75	0.681	0.439	0.293	1.002	0.995	0.049	0.339	0.267	0.787	0.90
D(2A-2C)	MLR	4	0.840	38.059	0.244	0.461	0.32-0.58	0.359	0.319	0.072	0.845	0.587	0.071	0.288	0.239	0.611	0.34
D(2A-2C)	PLS	3	0.865	59.134	0.219	0.362	0.16-0.61	0.277	0.299	0.111	0.832	0.570	0.159	0.299	0.251	0.559	0.30
D(2A-2C)	Ridge	12	0.906	18.037	0.220	0.484	0.33-0.62	0.361	0.403	0.096	0.906	0.567	0.065	0.264	0.222	0.631	0.44
D(2A-2C)	Lasso	11	0.869	17.285	0.235	0.511	0.37-0.58	0.407	0.353	0.082	0.970	0.562	0.077	0.272	0.230	0.622	0.56
D(2A-2C)	ElasticNet	16	0.889	9.120	0.253	0.524	0.41-0.63	0.456	0.406	0.080	0.979	0.566	0.050	0.268	0.224	0.657	0.60
D(2A-2C)	SVR	41	0.914	0.272	0.949	0.483	0.36-0.62	0.364	0.365	0.094	0.958	0.566	0.062	0.281	0.240	0.603	0.48
D(2A-2C)	RF	41	0.922	0.285	0.915	0.499	0.42-0.61	0.407	0.362	0.094	1.004	0.512	0.073	0.271	0.225	0.603	0.50
D(2A-2C)	XGB	41	1.000	2039.0	0.011	0.524	0.41-0.62	0.459	0.384	0.080	0.879	0.615	0.061	0.271	0.225	0.640	0.54
D(2A-2B)	MLR	4	0.784	26.293	0.237	0.374	-0.01-0.52	0.285	0.227	0.119	0.905	0.715	0.136	0.316	0.263	0.543	0.28
D(2A-2B)	PLS	4	0.869	50.122	0.184	0.509	0.31-0.63	0.466	0.391	0.151	0.974	0.765	0.064	0.252	0.219	0.706	0.52
D(2A-2B)	Ridge	14	0.913	14.121	0.187	0.509	0.40-0.65	0.482	0.374	0.174	1.007	0.751	0.032	0.250	0.214	0.674	0.52
D(2A-2B)	Lasso	14	0.900	13.144	0.197	0.485	0.24-0.63	0.425	0.356	0.175	0.991	0.753	0.028	0.261	0.222	0.686	0.46
D(2A-2B)	ElasticNet	17	0.911	9.296	0.217	0.484	0.26-0.63	0.442	0.356	0.172	0.970	0.757	0.025	0.262	0.228	0.668	0.48
D(2A-2B)	SVR	53	0.958	0.436	0.556	0.511	0.39-0.61	0.499	0.392	0.171	0.988	0.770	0.030	0.249	0.214	0.679	0.58
D(2A-2B)	RF	53	0.909	0.182	0.829	0.516	0.39-0.57	0.474	0.276	0.277	1.056	0.690	0.060	0.263	0.216	0.609	0.56
D(2A-2B)	XGB	53	1.000	2077.2	0.008	0.580	0.49-0.66	0.548	0.438	0.181	1.019	0.796	0.036	0.236	0.189	0.725	0.74

Columns as in Table S2. Because the partitions are numerically identical, this table can be read row by row against Table S2.

S9. Y-randomisation, PaDEL descriptors

Table S9. Response permutation on three partitions for each affinity endpoint, PaDEL descriptors.

Endpoint	Partition	Model	R2 real	Q2 real	R2 scr, median	Q2 scr, median	Q2 scr, max	cR2p	p
5-HT2A	lower quartile	PLS	0.926	0.615	0.423	-0.502	0.350	0.676	0.032
5-HT2A	lower quartile	Ridge	0.952	0.597	0.038	-0.021	0.124	0.864	0.032
5-HT2A	lower quartile	Lasso	0.973	0.645	0.000	0.000	0.278	0.926	0.032
5-HT2A	median	PLS	0.903	0.660	0.425	-0.765	0.333	0.652	0.032
5-HT2A	median	Ridge	0.917	0.688	0.030	-0.021	0.070	0.825	0.032

Endpoint	Partition	Model	R2 real	Q2 real	R2 scr, median	Q2 scr, median	Q2 scr, max	cR2p	p
5-HT2A	median	Lasso	0.930	0.584	0.000	0.000	0.281	0.895	0.032
5-HT2A	upper quartile	PLS	0.866	0.843	0.424	-0.191	0.513	0.620	0.032
5-HT2A	upper quartile	Ridge	0.882	0.836	0.017	-0.003	0.094	0.844	0.032
5-HT2A	upper quartile	Lasso	0.979	0.795	0.015	0.000	0.163	0.907	0.032
5-HT2B	lower quartile	PLS	0.898	0.801	0.402	-0.343	0.507	0.654	0.032
5-HT2B	lower quartile	Ridge	0.938	0.651	0.036	0.008	0.466	0.873	0.032
5-HT2B	lower quartile	Lasso	0.963	0.654	0.000	0.000	0.625	0.898	0.032
5-HT2B	median	PLS	0.889	0.651	0.422	-0.229	0.406	0.631	0.032
5-HT2B	median	Ridge	0.925	0.703	0.161	-0.018	0.105	0.822	0.032
5-HT2B	median	Lasso	0.933	0.687	0.000	0.000	0.272	0.884	0.032
5-HT2B	upper quartile	PLS	0.873	0.772	0.416	-0.271	0.564	0.630	0.032
5-HT2B	upper quartile	Ridge	0.899	0.760	0.020	-0.007	0.042	0.851	0.032
5-HT2B	upper quartile	Lasso	0.932	0.815	0.134	0.000	0.243	0.789	0.032
5-HT2C	lower quartile	PLS	0.908	0.642	0.423	-0.441	0.520	0.660	0.032
5-HT2C	lower quartile	Ridge	0.883	0.638	0.027	-0.005	0.337	0.836	0.032
5-HT2C	lower quartile	Lasso	0.943	0.581	0.000	0.000	0.229	0.864	0.032
5-HT2C	median	PLS	0.899	0.738	0.435	-0.719	0.566	0.652	0.032
5-HT2C	median	Ridge	0.933	0.746	0.046	-0.006	0.112	0.852	0.032
5-HT2C	median	Lasso	0.920	0.714	0.000	0.000	0.126	0.857	0.032
5-HT2C	upper quartile	PLS	0.898	0.803	0.461	-0.497	0.329	0.628	0.032
5-HT2C	upper quartile	Ridge	0.926	0.775	0.025	-0.003	0.314	0.875	0.032
5-HT2C	upper quartile	Lasso	0.986	0.765	0.043	0.000	0.098	0.882	0.032

Thirty permutations per model; 0.032 is the lower limit of the empirical p value with 30 permutations. Directly comparable to Table S3.

S10. Paired comparison of the two dictionaries

Table S10. Split-by-split comparison of the two descriptor dictionaries over identical partitions, with correction for multiplicity and a corrected resampled t test.

Endpoint	Model	SiRMS Q2F1	PaDEL Q2F1	Difference	PaDEL wins	Wilcoxon p	BH q	Holm p	t (NB)	p (NB)
5-HT2A	MLR	0.542	0.642	0.101	0.72	0.008	0.017	0.175	0.63	0.532
5-HT2A	PLS	0.696	0.714	-0.057	0.38	0.924	0.924	1.000	0.06	0.956
5-HT2A	Ridge	0.708	0.698	-0.068	0.40	0.540	0.635	1.000	-0.03	0.980
5-HT2A	Lasso	0.716	0.724	-0.016	0.48	0.592	0.676	1.000	0.16	0.871
5-HT2A	ElasticNet	0.712	0.704	-0.031	0.40	0.632	0.702	1.000	-0.04	0.966
5-HT2A	SVR	0.602	0.683	0.051	0.66	0.005	0.013	0.131	0.73	0.471
5-HT2A	RF	0.595	0.617	0.052	0.66	0.029	0.053	0.551	0.43	0.671
5-HT2A	XGB	0.585	0.699	0.113	0.74	0.001	0.002	0.015	0.84	0.406
5-HT2B	MLR	0.458	0.632	0.173	0.72	0.000	0.000	0.001	0.82	0.417
5-HT2B	PLS	0.652	0.704	0.100	0.64	0.005	0.013	0.131	0.63	0.534
5-HT2B	Ridge	0.658	0.698	0.069	0.60	0.143	0.212	1.000	0.37	0.714
5-HT2B	Lasso	0.719	0.697	-0.004	0.48	0.709	0.766	1.000	0.20	0.841
5-HT2B	ElasticNet	0.692	0.685	0.025	0.52	0.432	0.557	1.000	0.28	0.777
5-HT2B	SVR	0.559	0.663	0.091	0.78	0.000	0.000	0.001	0.91	0.365
5-HT2B	RF	0.484	0.636	0.137	0.92	0.000	0.000	0.000	1.33	0.190
5-HT2B	XGB	0.522	0.688	0.147	0.86	0.000	0.000	0.000	1.12	0.269

Endpoint	Model	SiRMS Q2F1	PaDEL Q2F1	Difference	PaDEL wins	Wilcoxon p	BH q	Holm p	t (NB)	p (NB)
5-HT2C	MLR	0.573	0.632	0.040	0.60	0.157	0.224	1.000	0.27	0.790
5-HT2C	PLS	0.692	0.719	-0.013	0.46	0.521	0.632	1.000	-0.34	0.739
5-HT2C	Ridge	0.803	0.724	-0.110	0.34	0.012	0.024	0.254	-0.60	0.553
5-HT2C	Lasso	0.779	0.649	-0.139	0.24	0.000	0.001	0.011	-0.87	0.386
5-HT2C	ElasticNet	0.799	0.688	-0.124	0.30	0.003	0.009	0.083	-0.74	0.463
5-HT2C	SVR	0.638	0.667	0.041	0.62	0.099	0.152	1.000	0.39	0.699
5-HT2C	RF	0.541	0.629	0.086	0.80	0.000	0.000	0.000	1.07	0.292
5-HT2C	XGB	0.653	0.693	0.037	0.64	0.034	0.059	0.606	0.44	0.659
D(2A-2C)	MLR	0.220	0.461	0.236	0.84	0.000	0.000	0.000	1.24	0.222
D(2A-2C)	PLS	0.394	0.362	-0.005	0.48	0.826	0.869	1.000	0.01	0.993
D(2A-2C)	Ridge	0.469	0.484	0.043	0.58	0.878	0.901	1.000	-0.11	0.913
D(2A-2C)	Lasso	0.446	0.511	0.051	0.62	0.053	0.085	0.848	0.24	0.815
D(2A-2C)	ElasticNet	0.445	0.524	0.137	0.62	0.018	0.036	0.379	0.36	0.720
D(2A-2C)	SVR	0.320	0.483	0.195	0.88	0.000	0.000	0.000	1.12	0.270
D(2A-2C)	RF	0.387	0.499	0.127	0.82	0.000	0.000	0.000	1.24	0.222
D(2A-2C)	XGB	0.394	0.524	0.154	0.72	0.000	0.000	0.000	1.20	0.236
D(2A-2B)	MLR	0.196	0.374	0.084	0.68	0.047	0.079	0.802	0.19	0.851
D(2A-2B)	PLS	0.395	0.509	0.099	0.62	0.027	0.051	0.537	0.46	0.649
D(2A-2B)	Ridge	0.493	0.509	0.021	0.54	0.461	0.576	1.000	-0.05	0.958
D(2A-2B)	Lasso	0.502	0.485	-0.012	0.46	0.169	0.233	1.000	-0.47	0.637
D(2A-2B)	ElasticNet	0.536	0.484	-0.010	0.48	0.318	0.424	1.000	-0.39	0.696
D(2A-2B)	SVR	0.422	0.511	0.093	0.62	0.001	0.003	0.031	0.88	0.386
D(2A-2B)	RF	0.336	0.516	0.151	0.84	0.000	0.000	0.000	1.28	0.205
D(2A-2B)	XGB	0.354	0.580	0.233	0.90	0.000	0.000	0.000	1.59	0.119

Medians over the same 50 partitions. BH q is the Benjamini-Hochberg adjusted value over all forty comparisons at a false discovery rate of 5 %; Holm p is the step-down adjusted value over the same family. The last two columns are the corrected resampled t test of Nadeau and Bengio, with the variance of the paired differences inflated by 1/k + n_test/n_train = 0.373; under that test no comparison reaches significance, which is the expected behaviour at this ratio of test-set to training-set size. The comparison is therefore reported as an effect size, with the median difference and the proportion of partitions favouring each dictionary as the primary measures.

S11. Scaffold-out partition, PaDEL descriptors

Table S11. Scaffold-out partition: training on the tryptamine series, testing on the 5-methoxytryptamine series, PaDEL descriptors.

Endpoint	Model	R^2tr	R^2te	CCC
5-HT2A	MLR	0.844	-11.949	0.145
5-HT2A	PLS	0.865	0.648	0.763
5-HT2A	Ridge	0.846	0.433	0.617
5-HT2A	Lasso	0.993	-0.652	0.400
5-HT2A	ElasticNet	1.000	0.367	0.667
5-HT2A	SVR	0.994	-1.059	0.029
5-HT2A	RF	0.920	-0.331	0.187
5-HT2A	XGB	1.000	-0.148	0.279
5-HT2B	MLR	0.872	0.581	0.718
5-HT2B	PLS	0.887	0.596	0.733
5-HT2B	Ridge	0.998	0.719	0.804

Endpoint	Model	R²tr	R²te	CCC
5-HT2B	Lasso	0.994	0.710	0.797
5-HT2B	ElasticNet	0.999	0.711	0.796
5-HT2B	SVR	0.990	-0.223	0.107
5-HT2B	RF	0.926	0.126	0.400
5-HT2B	XGB	1.000	0.042	0.495
5-HT2C	MLR	0.885	-0.198	0.510
5-HT2C	PLS	0.877	0.670	0.803
5-HT2C	Ridge	0.794	0.431	0.625
5-HT2C	Lasso	0.998	0.476	0.693
5-HT2C	ElasticNet	0.998	0.607	0.752
5-HT2C	SVR	0.959	-0.037	0.135
5-HT2C	RF	0.925	0.111	0.425
5-HT2C	XGB	1.000	0.048	0.496
D(2A-2C)	MLR	0.692	-0.092	0.539
D(2A-2C)	PLS	0.909	0.527	0.684
D(2A-2C)	Ridge	0.912	-0.006	0.518
D(2A-2C)	Lasso	0.864	0.013	0.493
D(2A-2C)	ElasticNet	0.864	0.017	0.494
D(2A-2C)	SVR	0.990	-7.667	0.005
D(2A-2C)	RF	0.859	0.231	0.458
D(2A-2C)	XGB	1.000	0.579	0.734
D(2A-2B)	MLR	0.835	-1.257	0.370
D(2A-2B)	PLS	0.896	0.258	0.688
D(2A-2B)	Ridge	0.913	0.200	0.659
D(2A-2B)	Lasso	0.965	-2.071	0.305
D(2A-2B)	ElasticNet	0.958	-0.681	0.484
D(2A-2B)	SVR	0.968	-0.912	0.100
D(2A-2B)	RF	0.866	0.124	0.402
D(2A-2B)	XGB	1.000	0.326	0.531

Directly comparable to Table S4. PLS on PaDEL descriptors is positive at all five endpoints, whereas the simplex models are negative at several.

S12. Position-out partition, PaDEL descriptors

Table S12. Position-out partitions: withholding the ortho, the meta and the para block in turn, PaDEL descriptors.

Withheld block	Endpoint	Model	R2te	Q2F1	RMSE	CCC	Outside AD	Mean AD ratio
ortho	5-HT2A	PLS	-0.4	-0.288	0.58	0.398	0%	0.57
ortho	5-HT2A	Ridge	-0.2	-0.091	0.53	0.473	0%	0.57
ortho	5-HT2A	Lasso	-0.6	-0.429	0.61	0.388	0%	0.57
ortho	5-HT2A	RF	-0.1	0.025	0.50	0.365	0%	0.57
ortho	5-HT2A	XGB	-0.5	-0.390	0.60	0.297	0%	0.57
ortho	5-HT2B	PLS	0.2	0.334	0.39	0.566	0%	0.68
ortho	5-HT2B	Ridge	0.2	0.345	0.38	0.564	0%	0.68
ortho	5-HT2B	Lasso	-0.6	-0.424	0.57	0.430	0%	0.68
ortho	5-HT2B	RF	0.3	0.380	0.37	0.538	0%	0.68

Withheld block	Endpoint	Model	R2te	Q2F1	RMSE	CCC	Outside AD	Mean AD ratio
ortho	5-HT2B	XGB	0.2	0.346	0.38	0.504	0%	0.68
ortho	5-HT2C	PLS	-0.9	-0.594	0.54	0.116	14%	0.73
ortho	5-HT2C	Ridge	-0.9	-0.599	0.54	0.103	14%	0.73
ortho	5-HT2C	Lasso	-1.4	-1.078	0.61	0.147	14%	0.73
ortho	5-HT2C	RF	0.2	0.283	0.36	0.418	14%	0.73
ortho	5-HT2C	XGB	-0.1	0.044	0.41	0.363	14%	0.73
ortho	D(2A-2C)	PLS	-2.4	-0.430	0.49	0.346	0%	0.27
ortho	D(2A-2C)	Ridge	-3.0	-0.646	0.53	0.361	0%	0.27
ortho	D(2A-2C)	Lasso	-5.7	-1.808	0.69	0.269	0%	0.27
ortho	D(2A-2C)	RF	-0.3	0.464	0.30	0.553	0%	0.27
ortho	D(2A-2C)	XGB	0.2	0.683	0.23	0.687	0%	0.27
ortho	D(2A-2B)	PLS	-0.3	0.527	0.29	0.521	0%	0.48
ortho	D(2A-2B)	Ridge	0.2	0.687	0.23	0.494	0%	0.48
ortho	D(2A-2B)	Lasso	-4.5	-1.080	0.60	0.168	0%	0.48
ortho	D(2A-2B)	RF	-0.5	0.432	0.31	0.436	0%	0.48
ortho	D(2A-2B)	XGB	-0.6	0.382	0.33	0.511	0%	0.48
meta	5-HT2A	PLS	-5.2	-0.193	1.31	0.192	0%	0.54
meta	5-HT2A	Ridge	-3.9	0.044	1.17	0.224	0%	0.54
meta	5-HT2A	Lasso	-3.0	0.230	1.05	0.270	0%	0.54
meta	5-HT2A	RF	-5.3	-0.227	1.33	0.141	0%	0.54
meta	5-HT2A	XGB	-5.4	-0.245	1.34	0.149	0%	0.54
meta	5-HT2B	PLS	-1.9	0.037	0.99	0.254	0%	0.52
meta	5-HT2B	Ridge	-2.1	-0.036	1.03	0.280	0%	0.52
meta	5-HT2B	Lasso	-0.9	0.388	0.79	0.440	0%	0.52
meta	5-HT2B	RF	-1.7	0.117	0.95	0.263	0%	0.52
meta	5-HT2B	XGB	-1.6	0.158	0.92	0.308	0%	0.52
meta	5-HT2C	PLS	-3.6	-0.152	0.98	0.225	0%	0.42
meta	5-HT2C	Ridge	-3.1	-0.034	0.93	0.237	0%	0.42
meta	5-HT2C	Lasso	-4.4	-0.346	1.06	0.161	0%	0.42
meta	5-HT2C	RF	-3.7	-0.169	0.99	0.162	0%	0.42
meta	5-HT2C	XGB	-4.5	-0.373	1.07	0.146	0%	0.42
meta	D(2A-2C)	PLS	-0.1	0.327	0.37	0.566	0%	0.16
meta	D(2A-2C)	Ridge	0.3	0.562	0.30	0.656	0%	0.16
meta	D(2A-2C)	Lasso	0.2	0.509	0.31	0.608	0%	0.16
meta	D(2A-2C)	RF	0.3	0.604	0.28	0.587	0%	0.16
meta	D(2A-2C)	XGB	0.4	0.643	0.27	0.707	0%	0.16
meta	D(2A-2B)	PLS	-0.8	0.125	0.33	0.462	0%	0.35
meta	D(2A-2B)	Ridge	-0.8	0.154	0.32	0.407	0%	0.35
meta	D(2A-2B)	Lasso	-0.7	0.164	0.32	0.239	0%	0.35
meta	D(2A-2B)	RF	-0.3	0.355	0.28	0.422	0%	0.35
meta	D(2A-2B)	XGB	-0.3	0.395	0.27	0.376	0%	0.35
para	5-HT2A	PLS	-11.0	-0.667	1.61	0.097	0%	0.37
para	5-HT2A	Ridge	-10.7	-0.630	1.59	0.096	0%	0.37
para	5-HT2A	Lasso	-13.2	-0.969	1.75	0.083	0%	0.37
para	5-HT2A	RF	-10.2	-0.552	1.55	0.091	0%	0.37
para	5-HT2A	XGB	-10.7	-0.621	1.58	0.099	0%	0.37
para	5-HT2B	PLS	-7.5	-0.019	0.73	0.205	0%	0.61

Withheld block	Endpoint	Model	R2te	Q2F1	RMSE	CCC	Outside AD	Mean AD ratio
para	5-HT2B	Ridge	-7.8	-0.063	0.75	0.204	0%	0.61
para	5-HT2B	Lasso	-13.0	-0.684	0.94	0.110	0%	0.61
para	5-HT2B	RF	-9.8	-0.302	0.83	0.199	0%	0.61
para	5-HT2B	XGB	-11.2	-0.472	0.88	0.214	0%	0.61
para	5-HT2C	PLS	-8.8	0.157	0.83	0.154	0%	0.57
para	5-HT2C	Ridge	-10.2	0.039	0.88	0.136	0%	0.57
para	5-HT2C	Lasso	-8.3	0.204	0.80	0.152	0%	0.57
para	5-HT2C	RF	-11.0	-0.029	0.91	0.103	0%	0.57
para	5-HT2C	XGB	-10.3	0.031	0.89	0.141	0%	0.57
para	D(2A-2C)	PLS	-1.9	-0.685	0.59	0.203	0%	0.18
para	D(2A-2C)	Ridge	-2.1	-0.802	0.61	0.193	0%	0.18
para	D(2A-2C)	Lasso	-2.3	-0.939	0.63	0.201	0%	0.18
para	D(2A-2C)	RF	-1.1	-0.237	0.50	0.284	0%	0.18
para	D(2A-2C)	XGB	-1.9	-0.661	0.58	0.233	0%	0.18
para	D(2A-2B)	PLS	-2.8	-0.484	0.74	0.154	0%	0.18
para	D(2A-2B)	Ridge	-2.7	-0.463	0.73	0.130	0%	0.18
para	D(2A-2B)	Lasso	-1.9	-0.149	0.65	0.158	0%	0.18
para	D(2A-2B)	RF	-1.6	-0.020	0.61	0.218	0%	0.18
para	D(2A-2B)	XGB	-1.7	-0.054	0.62	0.222	0%	0.18

Directly comparable to Table S7. The ordering of the three blocks is preserved at 5-HT2A and 5-HT2B and not at 5-HT2C, where the para block is the best predicted of the three; PaDEL transfers less well than the simplex dictionary to every withheld block.

S13. Fragment contributions from the two dictionaries

Table S13. Mean fragment contributions with standard deviations, by descriptor dictionary, receptor and partition scheme.

Fragment	SiRMS 2A	SiRMS 2B	SiRMS 2C	PaDEL 2A	PaDEL 2B	PaDEL 2C	SiRMS 2A (CV)	SiRMS 2B (CV)	SiRMS 2C (CV)	PaDEL 2A (CV)	PaDEL 2B (CV)	PaDEL 2C (CV)	n (CV)
N-benzyl	+0.820 (0.67)	-0.216 (0.45)	-0.950 (0.48)	+0.819 (0.63)	-0.241 (0.49)	-0.926 (0.49)	+0.841 (0.66)	-0.173 (0.46)	-0.903 (0.49)	+0.743 (0.68)	-0.191 (0.49)	-0.919 (0.52)	44
substituent	-0.359 (0.67)	+0.047 (0.45)	-0.310 (0.48)	-0.299 (0.63)	+0.031 (0.49)	-0.253 (0.49)	-0.326 (0.65)	+0.074 (0.47)	-0.238 (0.49)	-0.249 (0.62)	+0.008 (0.52)	-0.229 (0.50)	42
5-OMe	+0.606 (0.15)	+0.436 (0.13)	+0.332 (0.08)	+0.604 (0.10)	+0.402 (0.09)	+0.306 (0.09)	+0.618 (0.15)	+0.433 (0.12)	+0.331 (0.09)	+0.596 (0.11)	+0.400 (0.12)	+0.322 (0.13)	22

Values are in log units, given as mean (standard deviation) over the compounds in each fragment class. A positive value means that removing the fragment lowers the predicted affinity. The first six columns are the 50 repeated stratified splits of the original submission, which cover 105 of the 108 truncated structures; the columns marked CV are the twelve times repeated stratified four-fold cross-validation, under which every compound is held out twelve times and all 108 truncations are covered. The three truncations missing from the first scheme belong to the two unsubstituted benzyl analogues, which fall in strata of two members and therefore never enter a test partition.

Table S13b. Correlation of contribution profiles between receptors, by level, dictionary and partition scheme, with disattenuation.

Level and dictionary	Scheme	n	2A/2B	2B/2C	2A/2C	Reliability	2A/2C disattenuated
fragment, SiRMS	50 repeated splits	105	0.478	0.905	0.249	0.989 to 0.994	0.251

Level and dictionary	Scheme	n	2A/2B	2B/2C	2A/2C	Reliability	2A/2C disattenuated
fragment, SiRMS	repeated 4-fold CV	108	0.483	0.906	0.242	0.985 to 0.993	0.244
fragment, PaDEL	50 repeated splits	105	0.471	0.918	0.246	0.995 to 0.995	0.247
fragment, PaDEL	repeated 4-fold CV	108	0.555	0.890	0.285	0.956 to 0.983	0.295
atom, SiRMS	50 repeated splits	1692	0.639	0.723	0.290	0.950 to 0.953	0.305
atom, SiRMS	repeated 4-fold CV	1822	0.597	0.804	0.381	0.934 to 0.954	0.403
whole-molecule pKi	experimental	46	0.834	0.923	0.766	0.978 to 0.987	0.779

Reliability is the ratio of the variance between estimates to that variance plus the mean squared standard error of the individual estimates, and is given as the range over the three receptors. The disattenuated value is the observed correlation divided by the geometric mean of the two reliabilities. Because a contribution is a difference between two model predictions it carries model error twice, but the estimates are averaged over 12 to 50 models and are almost as reliable as the measured affinities; disattenuation therefore leaves the comparison between the fragment level and the whole-molecule level unchanged. The 5-HT2B/5-HT2C pair, computed from the same contributions by the same models, remains above 0.89 throughout.

S14. Stable PaDEL descriptors

Table S14. PaDEL descriptors selected by lasso in at least 40 % of the 50 repeats, with the direction of their coefficient.

Endpoint	Descriptor	Selection frequency	Mean sign
5-HT2A	JGI2	1.00	-1.00
5-HT2A	VE3_Dt	0.94	-1.00
5-HT2A	MATS4e	0.84	+1.00
5-HT2A	GGI3	0.80	+1.00
5-HT2A	ATSC5p	0.76	-1.00
5-HT2A	ATSC5s	0.64	+1.00
5-HT2A	MDEC-11	0.64	+1.00
5-HT2A	MATS6p	0.64	+1.00
5-HT2A	AATSC4e	0.58	+1.00
5-HT2A	minHBint5	0.56	-0.93
5-HT2A	SpMin1_Bhs	0.52	+1.00
5-HT2A	AATSC6p	0.42	+1.00
5-HT2A	GATS5p	0.42	+1.00
5-HT2A	VR2_Dzs	0.40	-1.00
5-HT2A	GATS5m	0.40	+1.00
5-HT2A	ATSC6c	0.40	-1.00
5-HT2B	ATSC5p	0.98	-1.00
5-HT2B	BCUTc-1h	0.98	-1.00
5-HT2B	JGI2	0.94	-1.00
5-HT2B	BIC3	0.90	+1.00
5-HT2B	ATSC6c	0.84	-1.00
5-HT2B	nHBint6	0.58	-1.00
5-HT2B	MDEC-11	0.52	+1.00
5-HT2B	GATS5i	0.48	+1.00
5-HT2B	BCUTw-1l	0.48	+1.00
5-HT2B	GATS6p	0.46	-1.00
5-HT2B	ATSC5s	0.46	+1.00
5-HT2B	MDEO-12	0.42	-1.00
5-HT2B	MATS5p	0.42	-1.00

Endpoint	Descriptor	Selection frequency	Mean sign
5-HT2B	MATS3s	0.40	-1.00
5-HT2C	GATS6p	0.82	-1.00
5-HT2C	JGI2	0.82	-1.00
5-HT2C	ATSC6c	0.76	-1.00
5-HT2C	AATSC5p	0.60	-0.93
5-HT2C	AATSC5s	0.58	+1.00
5-HT2C	ATSC5v	0.56	-1.00
5-HT2C	ALogp2	0.54	+1.00
5-HT2C	nHBint6	0.54	-1.00
5-HT2C	GGI7	0.52	-1.00
5-HT2C	GATS5i	0.50	+1.00
5-HT2C	BIC3	0.50	+1.00
5-HT2C	ATSC4e	0.48	+1.00
5-HT2C	MATS5p	0.48	-1.00
5-HT2C	GGI2	0.42	-1.00
5-HT2C	ATSC5m	0.40	-1.00

Complete selection frequencies are in padel_stability_pKi_5HT2A.csv, padel_stability_pKi_5HT2B.csv and padel_stability_pKi_5HT2C.csv. Unlike the simplex analysis, a small number of descriptors exceed 80 % selection frequency.

Supplementary figures

Figure S1. Observed versus out-of-fold predicted pKi from the PaDEL-based ridge models, with the applicability domain. Panels A to C: twelve repeats of stratified four-fold cross-validation; error bars are the standard deviation over the twelve predictions of each compound, the solid line is the identity and the dashed lines mark ±0.5 log units. Symbols encode benzyl substitution position and colours the scaffold. Panel D: standardised residual against k-nearest-neighbour distance to the training set normalised by the acceptance threshold. As with the simplex models, para-substituted compounds lie well inside the domain.

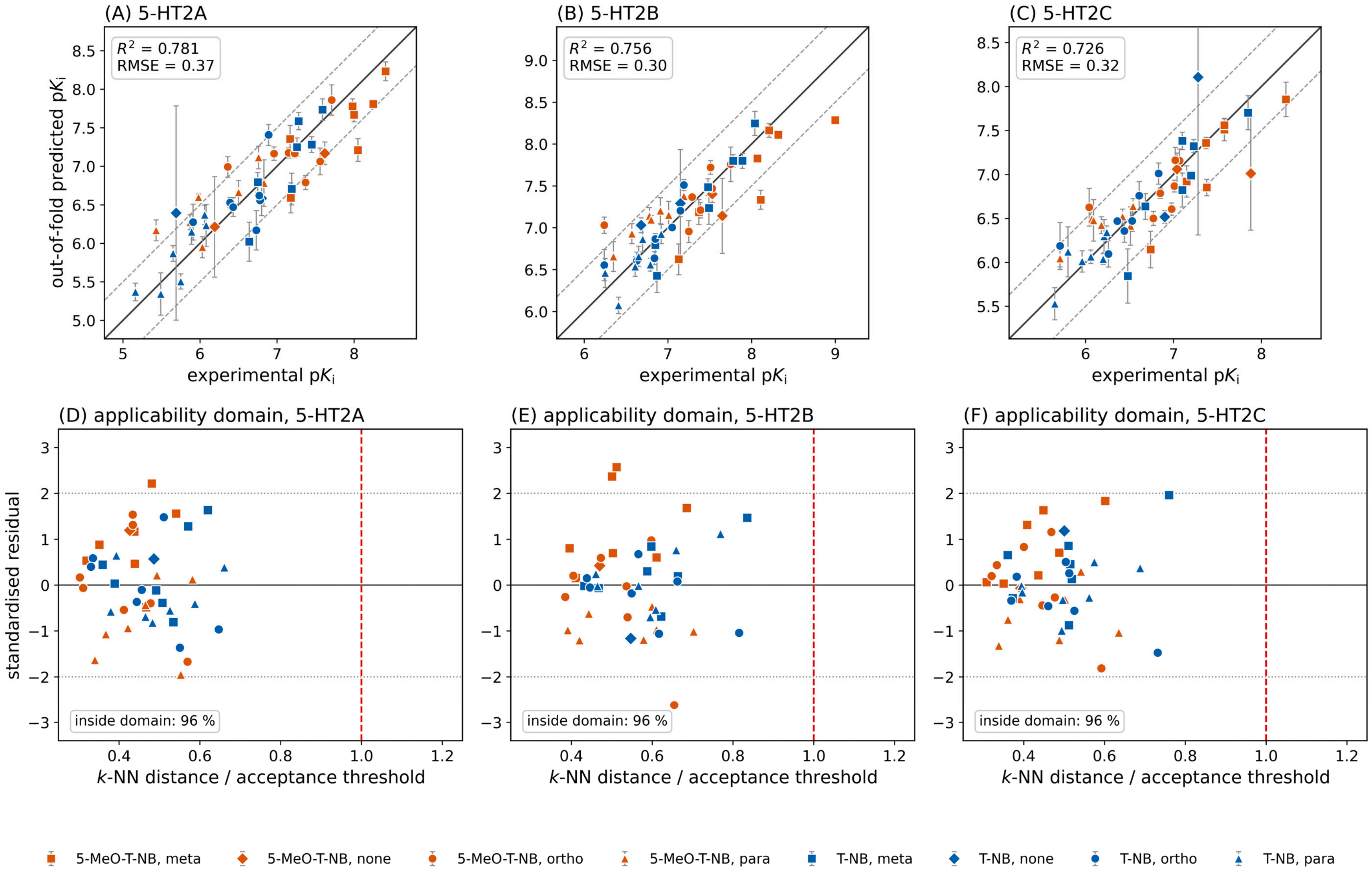

Figure S2. Transfer from the tryptamine series to the 5-methoxytryptamine series, for both descriptor dictionaries. Bars are the test-set coefficient of determination when models trained on the 23 tryptamine analogues are applied to the 23 5-methoxytryptamine analogues. Negative values indicate performance worse than predicting the training mean. PaDEL descriptors transfer more reliably across the scaffold change, because the 5-methoxy series contains fragment types absent from the simplex training set.

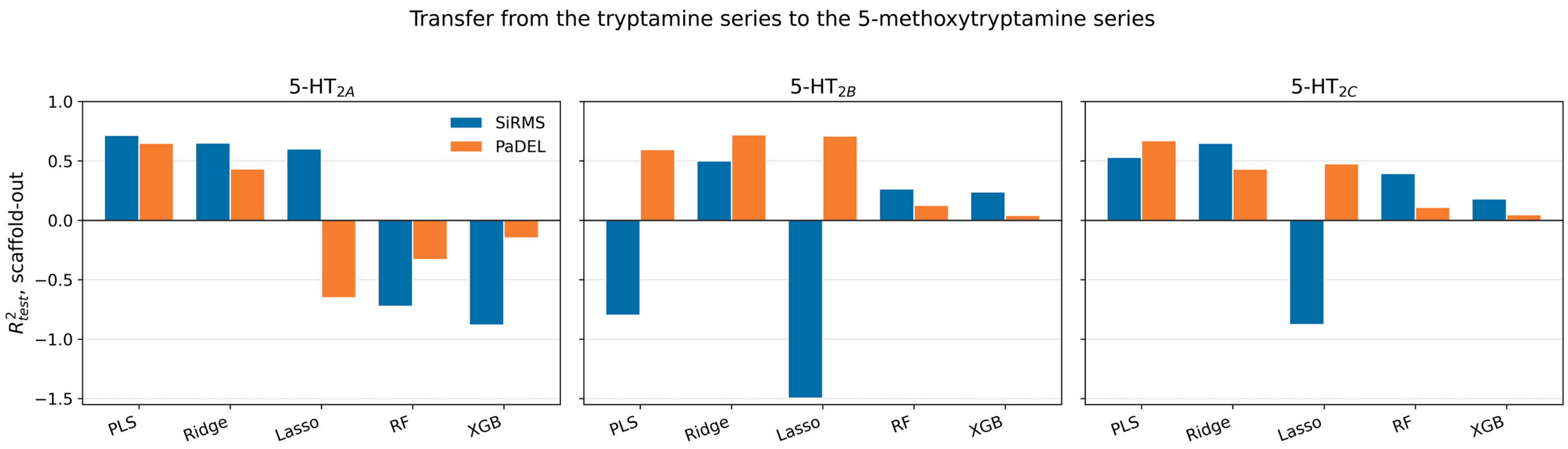

S15. Redundancy between the two descriptor dictionaries

Table S15. Numerical redundancy within and between the two descriptor dictionaries.

Comparison	Endpoint	n SiRMS	n PaDEL	Median highest |r|	% above 0.9	% above 0.95
raw dictionaries	all endpoints	1595	986	0.89	47	37
within SiRMS	all endpoints	1595	1595	1.00	93	84
within PaDEL	all endpoints	986	986	0.99	91	79
after filtering	pKi_5HT2A	106	143	0.81	25	10
after filtering	pKi_5HT2B	97	167	0.81	15	5
after filtering	pKi_5HT2C	97	143	0.79	16	4
stable selections	pKi_5HT2A (max |r| = 0.85)	12	16	0.68	0	0
stable selections	pKi_5HT2B (max |r| = 0.72)	8	14	0.62	0	0
stable selections	pKi_5HT2C (max |r| = 0.79)	11	15	0.61	0	0

For each descriptor the highest absolute Pearson correlation to any descriptor of the comparison set is taken over the 46 compounds, and the table reports the median of those values together with the proportion exceeding 0.9 and 0.95. No variable is shared between the dictionaries. Redundancy between them is lower than within either of them, which is expected when 46 molecules are described by more than a thousand variables. After training-set filtering, which determines what the models actually see, the overlap falls further, and among the descriptors selected by lasso in at least 40 % of repeats no cross-dictionary pair exceeds 0.9 at any receptor; the largest single values are 0.85, 0.72 and 0.79 at 5-HT2A, 5-HT2B and 5-HT2C. Descriptors with zero variance across the 46 compounds are excluded.

S16. Sensitivity to the size of the candidate descriptor pool

Table S16. Model performance as a function of the number of descriptors surviving univariate prescreening.

Dictionary	Endpoint	Model	Pool	Descriptors retained	R2tr	Q2F1	Q2F1 IQR	RMSE	CCC
PaDEL	5-HT2A	Lasso	300	134	0.956	0.724	0.60-0.81	0.427	0.842
PaDEL	5-HT2A	Lasso	100	54	0.940	0.673	0.60-0.76	0.467	0.818
PaDEL	5-HT2A	Lasso	50	32	0.908	0.675	0.55-0.74	0.466	0.807
PaDEL	5-HT2A	Lasso	25	17	0.840	0.665	0.53-0.72	0.492	0.774
PaDEL	5-HT2A	Ridge	300	134	0.935	0.698	0.59-0.78	0.442	0.825
PaDEL	5-HT2A	Ridge	100	54	0.920	0.690	0.61-0.76	0.450	0.819
PaDEL	5-HT2A	Ridge	50	32	0.892	0.672	0.58-0.74	0.464	0.801
PaDEL	5-HT2A	Ridge	25	17	0.836	0.645	0.51-0.71	0.491	0.775
PaDEL	5-HT2B	Lasso	300	165	0.948	0.697	0.61-0.78	0.349	0.809
PaDEL	5-HT2B	Lasso	100	63	0.935	0.659	0.53-0.75	0.359	0.816
PaDEL	5-HT2B	Lasso	50	34	0.908	0.661	0.58-0.75	0.373	0.797
PaDEL	5-HT2B	Lasso	25	19	0.875	0.632	0.52-0.71	0.357	0.764
PaDEL	5-HT2B	Ridge	300	165	0.919	0.698	0.63-0.77	0.341	0.819
PaDEL	5-HT2B	Ridge	100	63	0.911	0.675	0.54-0.75	0.355	0.803
PaDEL	5-HT2B	Ridge	50	34	0.893	0.668	0.59-0.74	0.353	0.800
PaDEL	5-HT2B	Ridge	25	19	0.843	0.631	0.50-0.73	0.372	0.766
PaDEL	5-HT2C	Lasso	300	127	0.958	0.649	0.43-0.75	0.352	0.805
PaDEL	5-HT2C	Lasso	100	60	0.944	0.674	0.55-0.76	0.375	0.813
PaDEL	5-HT2C	Lasso	50	38	0.927	0.671	0.55-0.75	0.349	0.811
PaDEL	5-HT2C	Lasso	25	20	0.901	0.635	0.45-0.69	0.388	0.766
PaDEL	5-HT2C	Ridge	300	127	0.926	0.724	0.62-0.77	0.342	0.818
PaDEL	5-HT2C	Ridge	100	60	0.912	0.708	0.58-0.76	0.331	0.811
PaDEL	5-HT2C	Ridge	50	38	0.893	0.690	0.59-0.74	0.348	0.799
PaDEL	5-HT2C	Ridge	25	20	0.878	0.630	0.44-0.71	0.375	0.756
SiRMS	5-HT2A	Lasso	300	105	0.964	0.716	0.59-0.80	0.422	0.846
SiRMS	5-HT2A	Lasso	100	48	0.925	0.693	0.56-0.77	0.439	0.822
SiRMS	5-HT2A	Lasso	50	29	0.890	0.665	0.53-0.74	0.454	0.811
SiRMS	5-HT2A	Lasso	25	16	0.858	0.616	0.55-0.73	0.486	0.765
SiRMS	5-HT2A	Ridge	300	105	0.948	0.708	0.61-0.81	0.405	0.847
SiRMS	5-HT2A	Ridge	100	48	0.920	0.707	0.61-0.77	0.430	0.822
SiRMS	5-HT2A	Ridge	50	29	0.849	0.651	0.58-0.74	0.455	0.797
SiRMS	5-HT2A	Ridge	25	16	0.807	0.625	0.53-0.71	0.490	0.759
SiRMS	5-HT2B	Lasso	300	97	0.959	0.719	0.48-0.82	0.332	0.842
SiRMS	5-HT2B	Lasso	100	49	0.900	0.562	0.40-0.70	0.395	0.741
SiRMS	5-HT2B	Lasso	50	30	0.862	0.467	0.27-0.59	0.473	0.683
SiRMS	5-HT2B	Lasso	25	16	0.736	0.433	0.30-0.56	0.456	0.650
SiRMS	5-HT2B	Ridge	300	97	0.941	0.658	0.54-0.77	0.350	0.792
SiRMS	5-HT2B	Ridge	100	49	0.898	0.557	0.37-0.68	0.413	0.720
SiRMS	5-HT2B	Ridge	50	30	0.846	0.485	0.21-0.59	0.459	0.676
SiRMS	5-HT2B	Ridge	25	16	0.705	0.428	0.29-0.52	0.493	0.610
SiRMS	5-HT2C	Lasso	300	94	0.985	0.779	0.63-0.89	0.253	0.878
SiRMS	5-HT2C	Lasso	100	52	0.962	0.691	0.55-0.80	0.321	0.836
SiRMS	5-HT2C	Lasso	50	28	0.938	0.660	0.56-0.76	0.336	0.824
SiRMS	5-HT2C	Lasso	25	16	0.848	0.591	0.41-0.70	0.372	0.750

Dictionary	Endpoint	Model	Pool	Descriptors retained	R2tr	Q2F1	Q2F1 IQR	RMSE	CCC
SiRMS	5-HT2C	Ridge	300	94	0.986	0.803	0.62-0.89	0.247	0.878
SiRMS	5-HT2C	Ridge	100	52	0.968	0.691	0.54-0.78	0.333	0.826
SiRMS	5-HT2C	Ridge	50	28	0.932	0.651	0.55-0.76	0.340	0.828
SiRMS	5-HT2C	Ridge	25	16	0.842	0.572	0.41-0.66	0.390	0.710

Medians over the same 50 stratified partitions of design B. The pool is the number of descriptors retained by univariate prescreening within each training partition before correlation pruning; 300 is the value used throughout the manuscript. Descriptors retained is the median number surviving both steps. External predictivity declines gradually as the pool is reduced twelve-fold, and the difference between R2tr and Q2F1 stays between 0.18 and 0.28 throughout, so the larger pool is not purchasing internal fit at the expense of external predictivity.

S17. Deterministic space-covering partition

Table S17. A Kennard-Stone partition of the same 46 compounds, evaluated against the distribution of the fifty stratified partitions of design B.

Dictionary	Endpoint	Model	Descriptors	R2tr	R2te	Q2F1	RMSE	CCC	design B median	design B IQR	percentile
SiRMS	5-HT2A	PLS	84	0.898	0.733	0.765	0.447	0.825	0.696	0.58-0.79	66
SiRMS	5-HT2A	Ridge	84	0.941	0.753	0.782	0.430	0.833	0.708	0.61-0.81	62
SiRMS	5-HT2A	Lasso	84	0.944	0.796	0.820	0.391	0.870	0.716	0.59-0.80	86
SiRMS	5-HT2B	PLS	90	0.915	0.768	0.771	0.234	0.867	0.652	0.50-0.76	76
SiRMS	5-HT2B	Ridge	90	0.944	0.772	0.775	0.232	0.854	0.658	0.54-0.77	76
SiRMS	5-HT2B	Lasso	90	0.951	0.797	0.800	0.219	0.882	0.719	0.48-0.82	72
SiRMS	5-HT2C	PLS	92	0.956	0.712	0.722	0.266	0.855	0.692	0.61-0.83	60
SiRMS	5-HT2C	Ridge	92	0.998	0.827	0.833	0.206	0.889	0.803	0.62-0.89	58
SiRMS	5-HT2C	Lasso	92	0.958	0.793	0.800	0.225	0.874	0.779	0.63-0.89	52
PaDEL	5-HT2A	PLS	135	0.885	0.837	0.840	0.303	0.902	0.714	0.59-0.78	88
PaDEL	5-HT2A	Ridge	135	0.942	0.781	0.785	0.351	0.870	0.698	0.59-0.78	78
PaDEL	5-HT2A	Lasso	135	0.949	0.718	0.723	0.399	0.830	0.724	0.60-0.81	48
PaDEL	5-HT2B	PLS	167	0.859	0.743	0.748	0.252	0.867	0.704	0.64-0.80	64
PaDEL	5-HT2B	Ridge	167	0.917	0.743	0.748	0.252	0.863	0.698	0.63-0.77	64
PaDEL	5-HT2B	Lasso	167	0.944	0.716	0.721	0.265	0.845	0.697	0.61-0.78	58
PaDEL	5-HT2C	PLS	141	0.641	-0.033	0.113	0.515	0.400	0.719	0.62-0.76	4
PaDEL	5-HT2C	Ridge	141	0.912	0.591	0.649	0.324	0.769	0.724	0.62-0.77	34
PaDEL	5-HT2C	Lasso	141	0.941	0.641	0.692	0.303	0.791	0.649	0.43-0.75	60

The Kennard-Stone algorithm selects the training set that covers the descriptor space most evenly, using no response information; it was applied separately within each dictionary, after removal of constant and collinear descriptors, to select 34 training compounds with the remaining 12 as the test set. The last column gives the position of the resulting single estimate within the distribution of Q2F1 over the fifty stratified partitions of the same algorithm and endpoint. With the simplex dictionary the split lies above the median in all nine combinations; with PaDEL it does so in six of nine and falls below 96 % of the random partitions in one. The two selections differ because the algorithm works on distances within its own descriptor space: the simplex test set contains six para-substituted, five meta-substituted and one ortho-substituted compound, the PaDEL test set five para, five ortho and two meta, and is dominated by halogen-substituted analogues.

Supplementary data files and scripts

All data files and scripts referred to here are contained in the deposited archive, which is organised as scripts/, data/ and intermediates/ and is described in its own README.

Data. dataset.xlsx (the 46 compounds with all endpoints and validation checks); sirms46_raw.csv and padel46_raw.csv (descriptor matrices); metrics_*.csv and padel_*.csv (per-split statistics for every algorithm and endpoint); sirms_vs_padel.csv and dictionary_comparison_corrected.csv (paired comparison, the latter with the Benjamini-Hochberg, Holm and corrected resampled t test values of Table S10); oof_predictions.csv and padel_oof.csv (out-of-fold predictions); scaffold_out.csv and padel_scaffold_out.csv (scaffold-out partition); position_out_blocks.csv (all three positional blocks, both dictionaries, Tables S7 and S12); prescreen_sensitivity.csv (Table S16); kennard_stone.csv (Table S17); yrand_three_splits.csv (Tables S3 and S9); dictionary_overlap.csv (Table S15); ablation_*.csv (labelling-scheme ablation); stability_*.csv and padel_stability_*.csv (selection frequencies); truncations.csv, fragcontrib_SiRMS_*.csv, fragcontrib_PaDEL_*.csv, atom_contributions_*.csv and contrib_scheme_comparison.csv (structural interpretation under both partition schemes, Tables S13 and S13b).

Scripts. paper_data.py and build_smiles.py (dataset generation and validation); sirms_2d.py and regen_intermediates.py (descriptor and truncated-structure matrices); qsar_pipeline.py and run_designB.py (partitions, filtering and modelling); metrics_full.py (validation statistics); padel_pipeline.py and padel_extra.py (second dictionary); position_out_blocks.py (positional partitions); prescreen_sensitivity.py (size of the candidate pool); kennard_stone.py (deterministic space-covering partition); yrand_three.py (response permutation on three partitions); ablation_stability.py (ablation and selection stability); frag_contrib.py, frag_both.py and frag_contrib_schemes.py (fragment and atomic contributions); oof_predictions.py (out-of-fold predictions); figure2_oof.py and figure3_atom_maps.py (Figures 2, 3 and S1).
